# Family shapes microbiome differences in Oklahoma salamanders

**DOI:** 10.3389/frmbi.2024.1277645

**Published:** 2024-03-13

**Authors:** Madelyn R. Kirsch, Sierra N. Smith, Daniel J. Becker, Jessa L. Watters, Katharine A. Marske, Cameron D. Siler, Hayley C. Lanier

**Affiliations:** ^1^Sam Noble Oklahoma Museum of Natural History, University of Oklahoma, Norman, OK, United States; ^2^School of Biological Sciences, University of Oklahoma, Norman, OK, United States

**Keywords:** *Batrachochytrium dendrobatidis*, conservation, disease, ecology, Plethodontidae, ranavirus, Salamandridae

## Abstract

**Introduction:**

Given the role of microbiomes in promoting host health and homeostasis, understanding the factors shaping skin microbial communities in wild vertebrates has become increasingly important in conservation. This goal is even more pressing for amphibians, for which the skin has multiple critical functions, and pathogens currently decimating populations are linked to significant changes in skin microbiomes. However, because microbiomes are also shaped by environmental and ecological influences, as well as by host phylogeny, it is important to quantify these contributions to microbiome structure in the presence of infection.

**Methods:**

To understand the joint influence of these diverse factors shaping microbiomes, we used 16S rRNA sequencing to characterize the skin microbial communities of six salamander species (families Plethodontidae and Salamandridae) found in Oklahoma and contrasted the effects of infection status, phylogeny, host ecology, and host environment (i.e., climate) on skin microbiomes.

**Results:**

Differences at the level of host family were the main factor influencing microbiome diversity; however, we did not detect a substantial phylogenetic signal. Instead, host ecology and environment were more important in driving microbiome differences among species and genera. Salamanders that tested positive for the skin fungal parasite *Batrachochytrium dendrobatidis* (*Bd*) also had slightly less diverse microbiomes than *Bd-free* animals, but no such differences were associated with the systemic pathogen ranavirus (RV).

**Discussion:**

Together, these results indicate a nuanced relationship between the number and type of microbes present on salamander skin and the factors influencing them. By developing a baseline assessment of the microbiome diversity and richness present on the skin of these focal species, this work also provides a foundation for monitoring and evaluating changes in skin microbiomes as populations continue to experience stressors and diseases.

## 1 Introduction

Microbiomes, the microorganisms or their genetic material that exist in a particular environment or on a host, are increasingly recognized as providing crucial functions relating to host health, aiding in processes such as digestion and protection from disease (Moloney et al., 2014). There is increasing recognition that even small changes in relative abundance within microbial communities can result in the loss of host innate functions (metabolic, physiological, and immune) – a condition referred to as dysbiosis–with profound negative effects on a host’s overall fitness (e.g., Chimenos-Küstner et al., 2017; Jiménez and Sommer, 2017). Dysbiosis can lead to behavioral and developmental changes as well as decreased immune function. For example, even a slight shift in the abundance or community structure of microbiota can be linked to illness across a wide range of taxa (e.g., humans, amphibians, insects; Hooper and Gordon, 2001; Brucker and Bordenstein, 2012; Kueneman et al., 2016; Chimenos-Küstner et al., 2017; Bletz et al., 2018). Thus, developing a baseline understanding of host-associated microbiomes and identifying the role of microbiomes in shaping susceptibility to infection is critical for many species, especially those of conservation concern (Jiménez and Sommer, 2017).

Amphibians are currently the most threatened vertebrates on the planet, with 41% of amphibian species threatened with extinction due to climate change, habitat loss, and infectious disease (IUCN, 2022). At the end of the twentieth century, two infectious diseases began to be linked to widespread declines in amphibian populations: chytridiomycosis (caused by *Batrachochytrium dendrobatidis* [*Bd*] or *B. salamandrivorans* [*Bsal*]) and ranavirus disease (caused by infection from one of the several viruses in the genus *Ranavirus* [family Iridoviridae]; Daszak et al., 1999; Lips et al., 2006; Fisher and Garner, 2020; Scheele et al., 2017; Skerratt et al., 2007). Amphibian susceptibility to *Bd* infection is influenced by their extremely permeable skin, which allows for cutaneous respiration and osmotic regulation (Campbell et al., 2012; Rollins-Smith, 2009; Smith et al., 2018). Vast microbial diversity is present on the skin of amphibian species, and certain microbial taxa can influence host immunity directly by producing antifungal or antimicrobial compounds (Bletz et al., 2017b; Woodhams et al., 2015). This has been observed with the presence of anti-*Bd* microbes on the skin of amphibians linking to reduced fungal infection, underscoring the important role beneficial bacteria may play in pathogen susceptibility among hosts (Rebollar et al., 2020; Mutnale et al., 2021; Nava-González et al., 2021). Amphibians vary in their susceptibility to pathogens (DiRenzo et al., 2021); this may be the result of both the host’s immune system and the varied microbial diversity found in the skin microbiome. Thus, it is important to characterize and compare microbial diversity across a range of species.

Complicating our understanding of the role amphibian microbiomes play in inhibiting infection is the fact that infection can change the composition of the skin microbiome (Wilber et al., 2020). Amphibians experience a significant reduction in the skin microbiome's diversity after recovery from chytridiomycosis (Jani and Briggs, 2014; Rebollar et al., 2016; Medina et al., 2017; Bates et al., 2019; Muletz-Wolz et al., 2019; Ruthsatz et al., 2020; Jani et al., 2021). Likewise, the skin microbiomes of amphibians that have experienced ranavirus infection exhibit a shift in composition compared to virus-free individuals or populations (Campbell et al., 2019; Harrison et al., 2019). Interestingly, the skin microbiomes of some amphibian taxa do not appear to be strongly impacted by fungal infection, such as that of the red-backed salamander (*Plethodon cinereus*; Becker and Harris, 2010; Barnes et al., 2021; Bates et al., 2022). Given the variation among these findings, additional studies documenting the diversity of microbes found in skin microbiomes and their relationship to infection are needed.

Fully understanding the relationship between amphibian infectious disease and host microbiomes requires first determining the baseline composition of the microbiome and the factors driving its diversity. Three main potential contributors to skin microbiomes have been identified—environment, ecology, and phylogeny—each with a unique set of predictions. If the physical environment (i.e., conditions related to local climate and/or biotic community) influences which microbes are able to colonize and thrive on a host, then amphibian species living in the same locality or ecoregion should show similar skin microbial composition. Past studies have found that abiotic aspects of the environment such as temperature, precipitation, and/or proxies for environmental similarity such as geographic distance (Ruthsatz et al., 2020; Walker et al., 2020) are significantly correlated with microbiome diversity. However, if host ecology (e.g., life stage or how a host interacts with the microclimates in its environment) is a greater predictor of skin microbial diversity than the animal’s surrounding environment, species’ skin microbiomes would differ based on a species’ ecological niche, developmental stage, and/or microhabitat preferences. Groups of amphibian species found in similar microhabitats (e.g., arboreal or aquatic) would exhibit similar skin microbiomes, regardless of geographical location. This could be due to lateral transfer among hosts or exposure to the same microhabitats or stressors (Bletz et al., 2017a). Thus, differences in specific microhabitat use, even within the same geographic region, could be significant enough to result in distinct skin microbiomes when compared to species in different niches. There is also evidence for host ecology driving skin microbial structure with differences in life history (Bletz et al., 2017a) and habitat type (Bird et al., 2018; Xu et al., 2020; Smith et al., 2023) shown to correlate significantly with skin microbial diversity.

In contrast to findings that implicate environment or host ecology as driving skin microbial diversity, multiple studies have found that phylogeny–i.e., differences related to either the evolutionary history or, as a proxy, taxonomic grouping reflective of that history–was the strongest predictor of diversity in amphibian skin microbiomes (McKenzie et al., 2012; Prado-Irwin et al., 2017). Certain microbes may be conserved as part of the skin microbiome due to evolutionary differences between amphibian species, and this could dictate which microbes become part of the microbiome. Among the few studies that have investigated skin microbial makeup in amphibians, sampling does not often involve a wide range of taxa, and where it does, phylogeny isn’t often explicitly considered (e.g., Ellison et al., 2019), or is considered by contrasting hosts at different taxonomic levels as a proxy for phylogenetic divergence (e.g., Buttimer et al., 2022). In a study on the salamander genera *Batrachoseps*, *Ensatina*, and *Taricha*, host genus was the main predictor of skin microbial richness (Buttimer et al., 2022). In contrast, a similar study focused only on *Batrachoseps* and *Ensatina* found that habitat was the most important factor shaping diversity (Bird et al., 2018). Thus, the taxonomic and phylogenetic sampling of a study, as well as the analytical approaches employed, may influence the inferred importance of factors shaping microbiome diversity. Occasions in which phylogenetic relationships appear to drive skin microbial diversity could also be an example of phylosymbiosis, in which the difference in skin microbiome makeup of two species may correspond to the evolutionary history and phylogenetic distance between the hosts (Lim and Bordenstein, 2020). Previous results are suggestive of the possible presence of phylosymbiotic relationships in amphibians (e.g., Kueneman et al., 2014; Ellison et al., 2019; Buttimer et al., 2022).

Ultimately, multiple drivers are likely to impact the diversity of skin microbiomes simultaneously. For example, a study considering multiple interacting factors in four frog and one newt taxa found that species identity correlated most strongly with microbial community composition differences, but habitat location was a secondary result of significant variation within each species (Kueneman et al., 2014). Similarly, a study of 49 frog (genus *Plectrohyla*) and 23 salamander (genera *Bolitoglossa* and *Pseudoeurycea*) species concluded that family, genus, or species identity (i.e., some aspect of the species’ ancestry; “phylogeny”) was the most important indicator of skin microbial diversity between different orders and families; however, between genera and species, microbial diversity was most impacted by host habitat (Ellison et al., 2019). Despite numerous studies, a consensus on the drivers of amphibian skin microbial diversity has not been reached. Thus, examining multiple interacting drivers simultaneously is necessary to understand how microbiomes assemble and how they intersect with amphibian disease.

**Figure 1:**
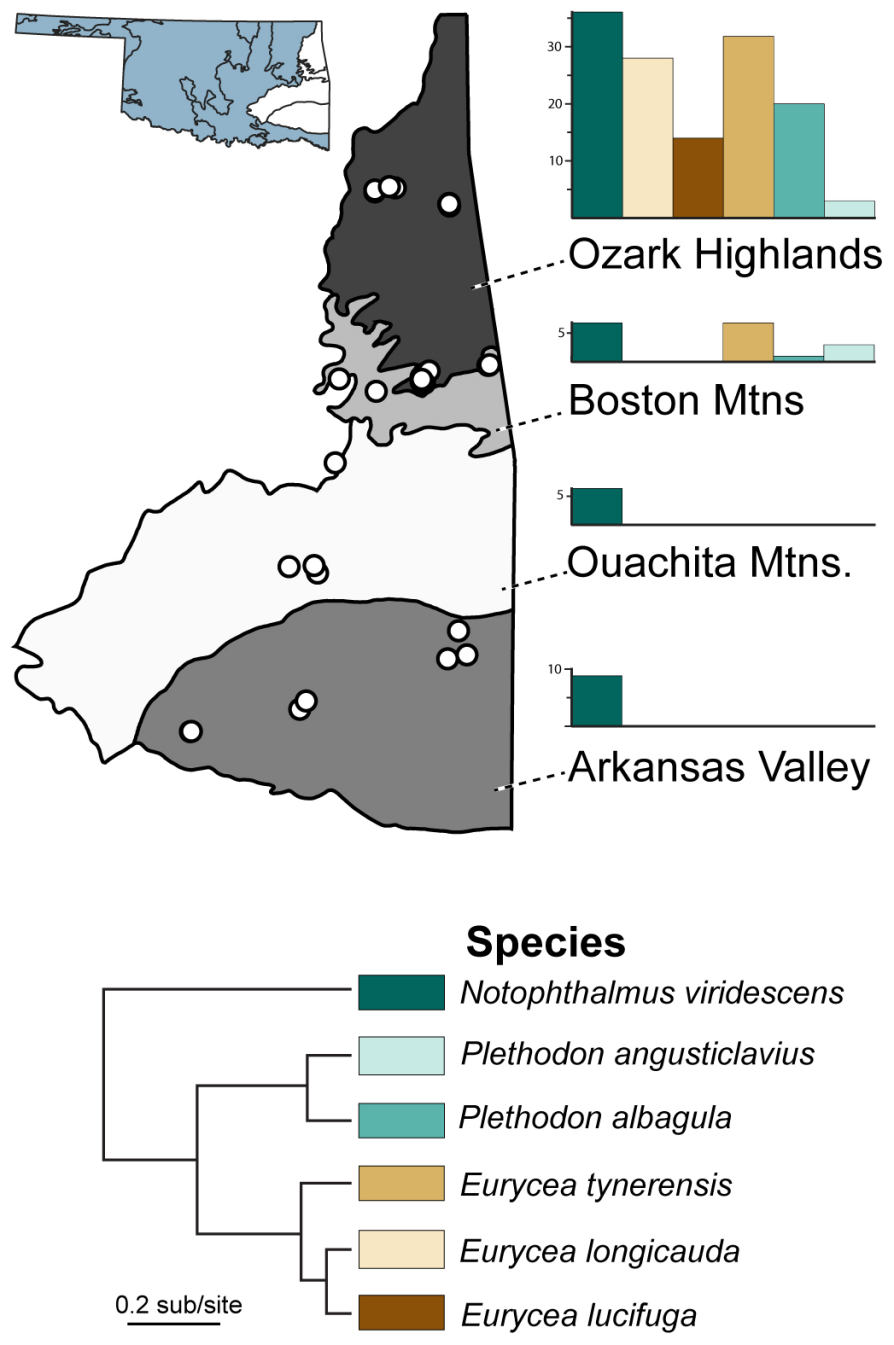
Sampling locations and abundance of six salamander host species in this study. Sampling localities (points) occur within the four level III ecoregions (Arkansas Valley, Boston Mountains, Ouachita Mountains, and Ozark Highlands) in eastern Oklahoma, USA. Bar charts refer to the proportion of salamander species found within each ecoregion.

In this study, skin microbiome data were collected from six focal, co-distributed species of salamanders representing two families and three genera across four level-III ecoregions (i.e., areas of distinct geography with co-distributed biota) in Oklahoma (**Figure 1**; **Supplementary Table 1**). As one of only four states in the country with 12 or more level-III ecoregions (Hoagland and Stoodley, 1998), Oklahoma is particularly well suited for comparisons among differing environments. In terms of host ecology, we sampled two direct-developing species (*Plethodon albagula* and *P. angusticlavius*; family Plethodontidae), three species with aquatic larvae and terrestrial or aquatic adults (*Eurycea lucifuga*, *E. longicauda*, and *E. tynerensis*; family Plethodontidae), and one species with an aquatic larval phase, terrestrial juvenile phase, and aquatic adult phase (*Notophthalmus viridescens*; family Salamandridae). Many of these samples were previously examined for pathogens (*Bd* and ranavirus), or we evaluated pathogen presence in the context of this study. Our goals were to (1) characterize the skin microbiomes of these six salamander species; (2) evaluate the role of the host’s environment (evaluated by both ecoregional differentiation and directly using climatic variables), host ecology, and evolutionary history on skin microbiome composition and similarity; and (3) determine the relationship between pathogen presence and microbial abundance and diversity. The results provide baseline knowledge about skin microbial composition in salamander species across regions of the United States for which little is known. Additionally, the findings add to a growing body of literature focused on the intersection of infectious disease with other factors structuring the skin microbiomes of vertebrates.

## 2 Methods

### 2.1 Sample populations

Here, we used epithelial swab samples and pathogen data, some with associated museum-vouchered specimens, collected by the Herpetology Department of the Sam Noble Oklahoma Museum of Natural History (SNOMNH) from 2015–2021 (Marhanka et al., 2017; Watters et al., 2018; Davis et al., 2019; Watters et al., 2019). We identified 287 samples (**Supplementary Data**) representing six species of salamanders with sufficient sampling for robust characterization of variation in species-specific skin microbiomes: *Eurycea longicauda, E. lucifuga*, *E. tynerensis*, *Plethodon albagula* and *P. angusticlavius*, and *Notophthalmus viridescens*. Samples were selected from four unique Level III ecoregions across eastern Oklahoma: Arkansas Valley, Boston Mountains, Ouachita Mountains, and Ozark Highlands (**Figure 1**; Hoagland and Stoodley, 1998). Where available, pathogen status (i.e., presence/absence of *Bd* and ranavirus) was ascertained from prior amphibian surveys containing published pathogen data (Marhanka et al., 2017; Watters et al., 2018; Davis et al., 2019; Watters et al., 2019). Samples that had not been previously assayed for pathogens were analyzed as described below.

### 2.2 Sample collection

During each collection effort (comprising 24–72 hours each), salamanders were captured in ponds, wetlands, and streams by hand, dipnet, aquatic trap, or seine. Animals were kept individually in sterile plastic bags before being swabbed and either released or euthanized and vouchered in the Herpetology Collection at SNOMNH. Microbiome samples were taken by rubbing a cotton swab tip (Puritan Medical Products, Orlinda, Tennessee, United States) along the ventral and dorsal portions of the trunk, limbs, and toes five times each to collect microbial DNA, and the animal was swabbed in the same way to test for the presence of Bd, using rayon-tipped sterile swabs (Peel Pouch Dryswab Fine Tip [MWE 113], Corsham, Wiltshire, UK), following the methods of Lannoo et al. (2011). Microbial swabs were placed into empty vials and flash-frozen using liquid nitrogen prior to long-term storage at –20°C. Swab samples for *Bd* were placed in a dry ice cooler prior to long-term storage at –20°C. Animals were sampled for ranavirus by either i) clipping off a portion of the tip of the tail, or ii) euthanization by chlorobutanol or MS-222 solution submersion and then dissection to obtain a tissue sample from the liver, following St-Amour and Lesbarrères (2007) and Gray et al. (2012). Tissue samples were then placed in long-term storage at 20°C. All sampling was conducted in accordance with University of Oklahoma IACUC Protocols R14-026 (2015–2017), R17-031 (2017–2020), and R21-005 (2020–2023).

### 2.3 Life history stage and host ecology

The life history stage was assessed during sampling or determined from examination of vouchered specimens at the SNOMNH. Individuals were classified as adults if the snout–vent length (SVL) was greater than or equal to 31 mm for *E. longicauda*, 26 mm for *E. tynerensis*, 55 mm for *P. albagula*, and 30 mm for *P. angusticlavius* (Trauth et al., 2004). *Eurycea lucifuga* were considered adults if the total length (TL) of the body was 100 mm or greater (Petranka and Scott, 1998). Individuals from either species of *Plethodon* were classified as juveniles if below the adult threshold SVL values listed above. Members of *Eurycea* were considered larval if they were below the SVL or TL adult thresholds and had gills; they were classified as juveniles if they had no external gills but were below the length requirement. Adults belonging to *E. tynerensis* were determined to be paedomorphic if external gills could be seen and they were above the length threshold. All vouchered newts in the SNOMNH Herpetology Collection were adults; they were distinct from larval or eft forms, based upon comparison to published images and descriptions (Trauth et al., 2004).

Samples were labeled as coming from terrestrial or aquatic individuals based on the age and physical characteristics of the animal. If gills were present in the *Eurycea*, samples were listed as coming from aquatic individuals. Since all newts were determined to be adults, they were listed as aquatic (Trauth et al., 2004). All other animals were categorized as terrestrial.

### 2.4 Climate and elevation data

Latitude and longitude were recorded for each sample at the time of sample collection using WGS 84. Using these coordinates, elevation was retrieved using an online map tool (The National Map; U. S. Geological Survey, 2017). Abiotic environmental conditions on the local climate—average annual, seasonal, and monthly precipitation and temperature data—associated with sample coordinates and month/year of sampling were assessed for each locality using ClimateNA (Wang et al., 2016). Before use in analyses of alpha and beta diversity, environmental data were centered, with a mean of zero, and scaled, to normalize standard deviations, in *R* software version 4.2.1 (R Core Team, 2022).

### 2.5 DNA Extraction and Sequencing

Genomic DNA from epithelial samples was extracted in the Sam Noble Museum Genomics Core Facility using ZymoBIOMICS DNA Miniprep Kits (Zymo Research Products, Irvine, CA, United States). Extraction negatives were run with each extraction, and PCR negative and positive controls were run on each PCR plate. Positive controls consisted of the ZymoBIOMICS Microbial Community Standard and the ZymoBIOMICS Microbial Community DNA Standard (Zymo Research Products, Irvine, CA, United States, RRID : SCR_008968). The V4 subregion of the 16S rRNA gene was amplified using a one-step PCR with the barcoded primers and protocols described in Kozich et al. (2013). We visualized 2 µL of several samples per plate via gel electrophoresis to ensure amplification, after which all samples were cleaned using KAPA Pure Beads (Roche Sequencing Solutions, Pleasanton, CA, United States). DNA concentrations were determined using a Qubit Fluorometer (Thermo Fisher Scientific, Waltham, MA, United States), and samples were normalized to 10 nM of DNA before pooling all samples in a 1.5 mL sterile microcentrifuge tube. The pooled library was then sequenced through a single run on an Illumina MiSeq platform (RRID : SCR_016379) using 2 × 250 bp paired-end sequencing at the University of Oklahoma Consolidated Core Lab.

Pathogen presence data were sourced from previous surveys (Marhanka et al., 2017; Watters et al., 2018; Davis et al., 2019). Recent samples that had not yet been evaluated for pathogens were processed following the methods of Watters et al. (2018). In brief, DNA was extracted using PrepMan Ultra (*Bd* assays; Life Technologies, Carlsbad, CA, United States; Cheng et al., 2011) and/or a method of high-salt DNA extraction (ranavirus assays; Esselstyn et al., 2008). Pathogens were amplified following previously published qualitative PCR procedures (qPCR; Kerby et al., 2013). Primers targeted the internal transcribed spacer (ITS-1) ribosomal RNA gene (forward primer: ITS1-3 Chytr; reverse primer: 5.8S) for *Bd* and the major capsid protein (MCP) for ranavirus (Boyle et al., 2004; Forson and Storfer, 2006). All samples were run in triplicate, and any samples with positive values <1.0 gene copies and/or only one well testing positive were run a second time.

### 2.6 Analysis

Paired-end sequencing reads were trimmed to a minimum length of 30 and aligned using AdapterRemoval v.2 (RRID : SCR_011834, Schubert et al., 2016). Chimeras were removed and non-chimeric sequences were clustered into Amplicon Sequence Variants (ASVs) using VSEARCH v.2.21.1 (Rognes et al., 2016). Amplicon sequence variants were classified against the EzTaxon database (Yoon et al., 2017) and the antifungal isolates database compiled by Woodhams et al. (2015). If an ASV was a 99% or greater match to the EzTaxon database, it was identified to species; 94.5–99% matches were identified to genus and matches that were 88–94.5% identical were identified to family. We included only matches of 99% or above to the antifungal database, following the methods of Barnes et al. (2021). Sequences were aligned with MAFFT (Katoh, 2002). The resulting ASV table, taxa table, metadata, and phylogenetic tree of microbes were imported into *R* and combined into a phyloseq object using the package *phyloseq* (RRID : SCR_013080, McMurdie and Holmes, 2013). Samples were rarefied to 200, 500, 1000, and 2000 reads per sample; results listed in the main text refer to rarefaction at 1000 reads (**Supplementary Figure 1**), a threshold chosen to remove samples with low sequence counts (Weiss et al., 2017).

We calculated three alpha diversity metrics (observed ASVs, Shannon-Wiener Index, and Inverse Simpson’s Index) using the *R* package *phyloseq*. These metrics were chosen to provide a range of complimentary insights into the microbiome. For example, observed ASVs provide the number of unique sequence variant clusters in a sample—a direct measure of taxonomic diversity. In contrast, the Shannon-Wiener Index accounts for evenness in addition to the number of distinct taxa, reducing the impact of rare variants. Finally, the Inverse Simpson’s Index accounts for species richness and evenness but weights microbe species by abundance, minimizing the impact of rare taxa. Shannon-Wiener and the Inverse Simpson’s indices are slightly more sensitive to species richness and species evenness, respectively (Johnson and Burnet, 2016). Alpha diversity was analyzed using Kruskal-Wallis tests for explanatory variables with only two qualitative responses and a pairwise Wilcoxon rank-sum test with the Holm *p*-value correction to compare the remaining qualitative variables.

Because the factors shaping microbiome diversity likely act simultaneously, we examined the combined effects of the phylogenetic, environmental, and ecological explanatory variables using two complementary approaches. First, we applied a Classification and Regression Tree (CART) framework (R package *partykit*; Hothorn and Zeileis, 2015), with family, genus, and species included as categorical variables alongside additional predictors. The CART analysis was performed using the conditional inference tree command (ctree) with the number of input variables per tree randomly sampled and no restrictions applied to the number of splits in the tree. Second, we used a phylogenetic generalized linear mixed model (PGLMM; *R* packages *brms*, *geiger*, and *PhyloOrchard*; O’Meara et al., 2013; Pennell et al., 2014; Bürkner, 2017) to analyze alpha diversity while accounting for phylogenetic non-independence using a phylogeny based upon maximum likelihood inference of 12-genes (12712 bp) for 2871 species from Pyron and Wiens (2011). We used only the species relevant to our study in the model. To prepare data for the PGLMM, continuous explanatory variables were tested for collinearity and removed if they had a variance inflation factor (VIF) greater than four, which represents a conservative metric for minimizing covarying explanatory data (Johnston et al., 2018). Mean annual temperature and mean seasonal precipitation were not included in the model due to VIF > 4. The PGLMM was run with two random effects (phylogeny and species), ten chains, 20,000 iterations (thinned every 10 samples), and a Gaussian response. Phylogenetic signal (h2) was estimated from PGLMMs as the proportional variance explained by phylogeny, using intercept only models (with reduced iterations). The CART and PGLMM analyses were run on each alpha diversity index separately to quantify and contrast the most important drivers of alpha diversity.

To understand differences in abundance and beta diversity microbial community composition was evaluated in the program QIIME2 (RRID : SCR_008249, Bolyen et al., 2019). To identify specific microbes that were more abundant between different families or in the presence/absence of infection, differential abundances were calculated using the packages *DEseq2* and *ANCOM-BC* in *R* (Love et al., 2014; Lin and Peddada, 2020). To understand differences among microbial communities and the factors driving them, beta diversity metrics were calculated using the UniFrac command to obtain unweighted- and weighted-UniFrac distances in the *phyloseq* package (Lozupone and Knight, 2005). The unweighted test evaluates the presence and absence of ASVs, whereas the weighted test accounts for the abundance of ASVs. Both tests take phylogenetic distance into account. We generated a phylogeny for our microbial taxa by aligning data with MAFFT (Katoh, 2002) and using the Maximum-Likelihood algorithm implemented in FastTree (v2, Price et al., 2010) with a Jukes-Cantor + CAT model of nucleotide evolution. Beta diversity was analyzed using Permutational Multivariate Analysis of Variance tests on UniFrac results (PERMANOVA; Anderson, 2001) with Holm *p*-value correction and permutations set to 10,000. To investigate how beta diversity changes by climate while controlling for geographic distance, we conducted partial Mantel tests using the Bray-Curtis matrix of beta diversity values and a geodesic distance matrix and controlled for the geographic distance using the Euclidean distance between sample locations with the *R* packages *vegan* and *ecodist* (RRID : SCR_011950, Goslee and Urban, 2007; Oksanen et al., 2022).

## 3 Results

The initial dataset consisted of 287 epithelial swab samples (**Supplementary Data**). After sequencing and quality filtering, there were 280 samples with adequate sequencing depths. In total, 5,256,288 sequences were obtained from these 280 samples, with a total of 13,038 ASVs. The total number of ASVs present in each sample varied widely, from 1 to 429,627, with an average of 11,883 ASVs ± 3,252. This yield is comparable to the number of sequences in other studies on amphibian microbiomes (Muletz-Wolz et al., 2018; Buttimer et al., 2022; 2019). To adjust for differences in ASV sample sizes, samples were rarefied to 1000 reads per sample (**Supplementary Figure 1**; **Supplementary Table 1**). After rarefaction, 179 samples remained, with the number of unique ASVs ranging from 22–610 (average 103; **Supplementary Data**). Of these, 147 animals were tested for *Bd* presence (59 positive and 88 negative) and 151 were tested for ranavirus (21 positive and 130 negative; 26 were determined from tail clips, 122 from liver samples, one from both liver and tail clip samples, and two from either tail clip or liver samples), resulting in disease status data for both pathogens for 146 animals (see **Supplementary Data**).

**Figure 2:**
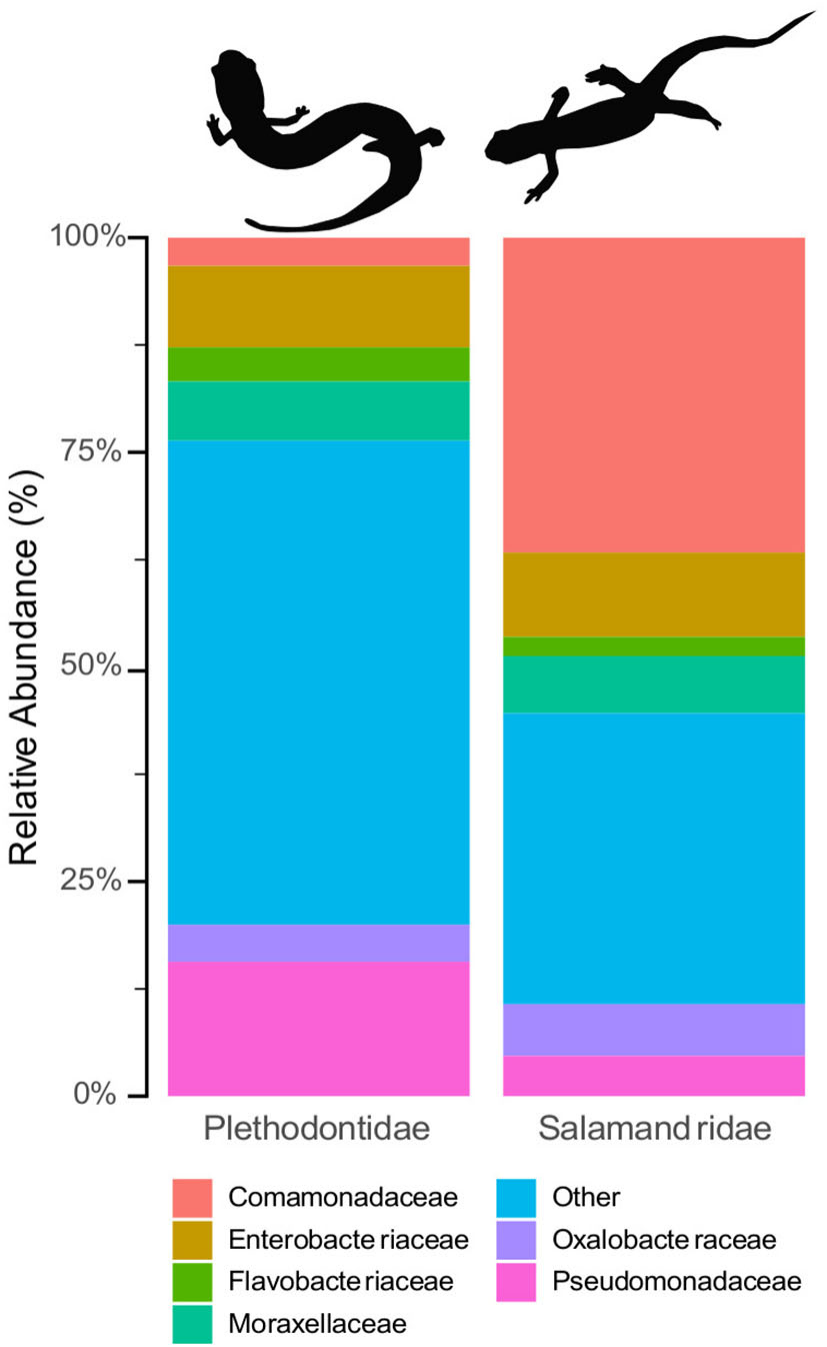
Relative compositional differences in skin microbiome communities based on host family. Plethodontids (left) had consistently higher alpha diversity than the Salamandridae (i.e., newts, right) and were distinct from the newts in analyses of beta diversity.

Hereafter, results refer to the final quality filtered dataset. Animals in the family Salamandridae (*n* = 71) had a skin microbial community composition of 37.3% Comamonadaceae, 10% Enterobacteriaceae, 7% Moraxellaceae, 6% Oxalobacteraceae, 5% Pseudomonadaceae, and 2% Flavobacteriaceae; 34% of the remaining microbes belonged to families that comprised less than 1% of the community (**Supplementary Figures 2, 3**). The majority of the microbial community on animals in family Plethodontidae (*n* = 108) was composed of microbial families that made up less than 1% of the community, which combined made up 56% of the microbiome. Plethodontids had a remaining skin microbial composition of 16% Pseudomonadaceae, 9% Enterobacteraceae, 7% Moraxellaceae, 4% Flavobacteraceae, 4% Oxalobacteraceae, and 3% Comamonadaceae (**Figure 2**; **Supplementary Figure 2, 3**). There were 79 differentially abundant microbes identified between the Plethodontidae and Salamandridae families by *DESeq2* and 79 microbes identified by the *ANCOM*-*BC*, with 28 microbial taxa identified by both analyses (**Supplementary Tables 3, 4**).

### 3.1 Alpha diversity

#### 3.1.1 Relationship to phylogenetic divergence among hosts

When contrasting alpha diversity by taxonomic grouping, without considering other explanatory variables, the family Plethodontidae had consistently higher microbial diversity than Salamandridae across all three metrics: observed ASVs, Shannon-Wiener Index, and Inverse Simpson’s Index (significance assessed with Kruskal-Wallis tests; **Supplementary Table 4**). This same pattern was observed between genera and species, with plethodontid species generally exhibiting greater alpha diversity than newts across all metrics; however, plethodontids were not significantly different from one another at the level of host genus or species (**Supplementary Figure 4**; **Supplementary Tables 4–6**).

**Figure 3:**
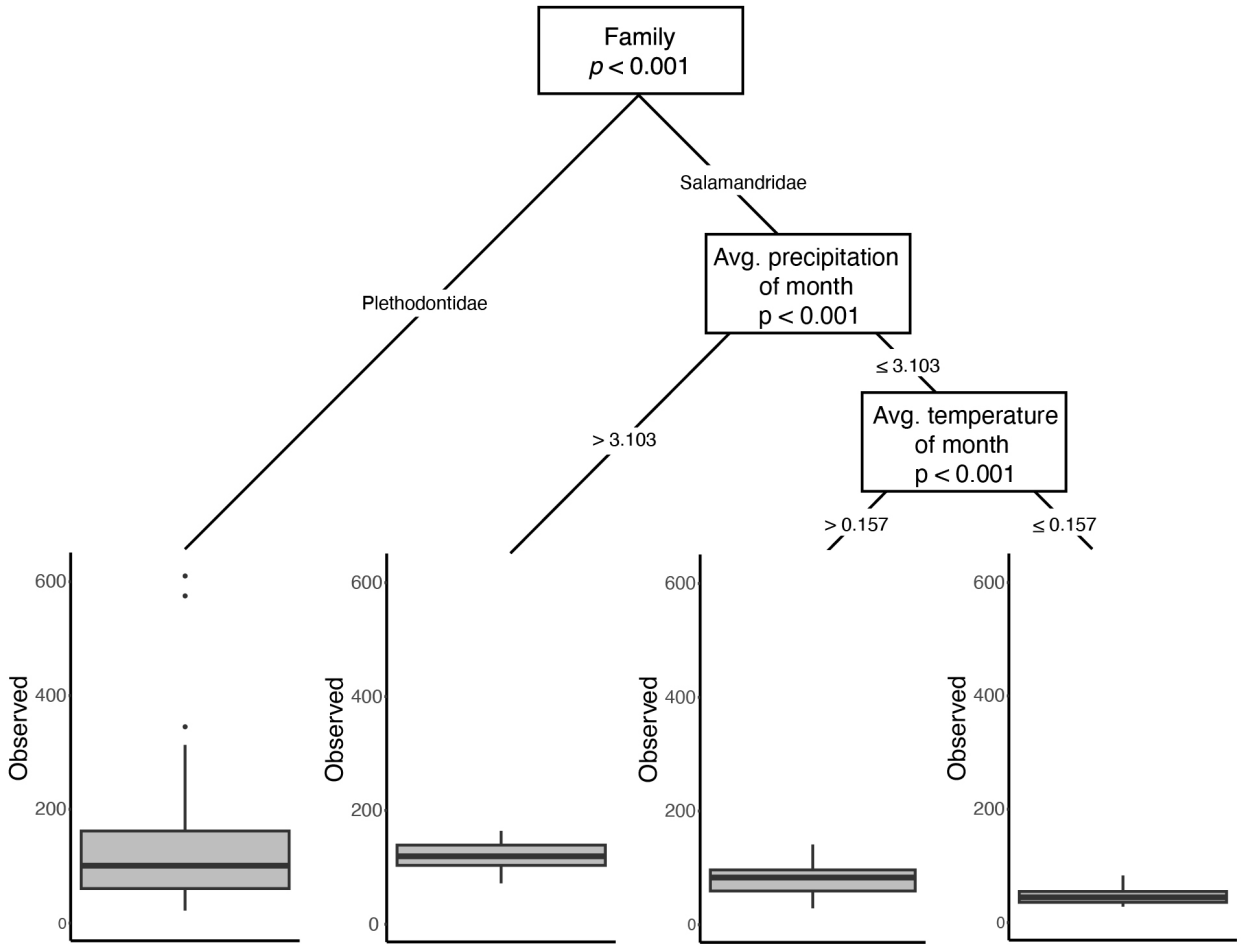
Results from the CART analysis of Observed ASVs. While family is the most important factor influencing the number of unique ASVs, environmental variables are important within family Salamandridae. Similar dominance of differentiation in microbial diversity based upon host phylogeny is observed in the other two metrics (**Supplementary Figures 2, 3**).

When host taxa were evaluated in the context of other explanatory variables in our CART analyses, a similar split was evident for both observed ASVs and the Shannon-Wiener Index: family-level divergence was the predominant split in the dataset (**Figure 3**; **Supplementary Figure 5**). For the Inverse Simpson’s Index, the primary factor that categorized microbiome diversity was related to species, with *E. lucifuga* and *P. angusticlavius* differing from all other taxa, followed by differences between plethodontids and newts (**Supplementary Figure 5**). However, we found low phylogenetic signal using PGLMMs [ASVs: h^2 ^= 0.01 (0.0–0.05 credible interval); Shannon: h^2 ^= 0.01 (0.0–0.03 credible interval); and Inverse Simpson: h^2 ^= 0.01 (0.0–0.06 credible interval)]. Despite detecting family-level differences in alpha diversity there was no evidence for phylogenetic signal below the level of family (i.e., at the level of genera or species). We found insufficient evidence for a phylogenetic signal for skin microbial diversity, despite detecting family-level differences.

#### 3.1.2 Relationship to environmental differences among hosts

For univariate analyses of the effect of ecoregion on microbiome diversity, we subdivided the data by host family and only contrasted species present in more than one ecoregion. Newts were present in all four ecoregions, but ecoregion was not a significant predictor of skin microbiome diversity for all comparisons in all alpha diversity metrics (**Supplementary Table 5**). We evaluated the ecoregion in plethodontids by excluding species that were not present in multiple ecoregions, resulting in three species present in two ecoregions (Ozark Highlands and the Boston Mountains; **Figure 1**; **Supplementary Table 1**). Across these three species (combined), individuals from the Ozark Highlands had significantly more microbial ASVs than those from the Boston Mountains (**Supplementary Table 6**).

**Figure 4:**
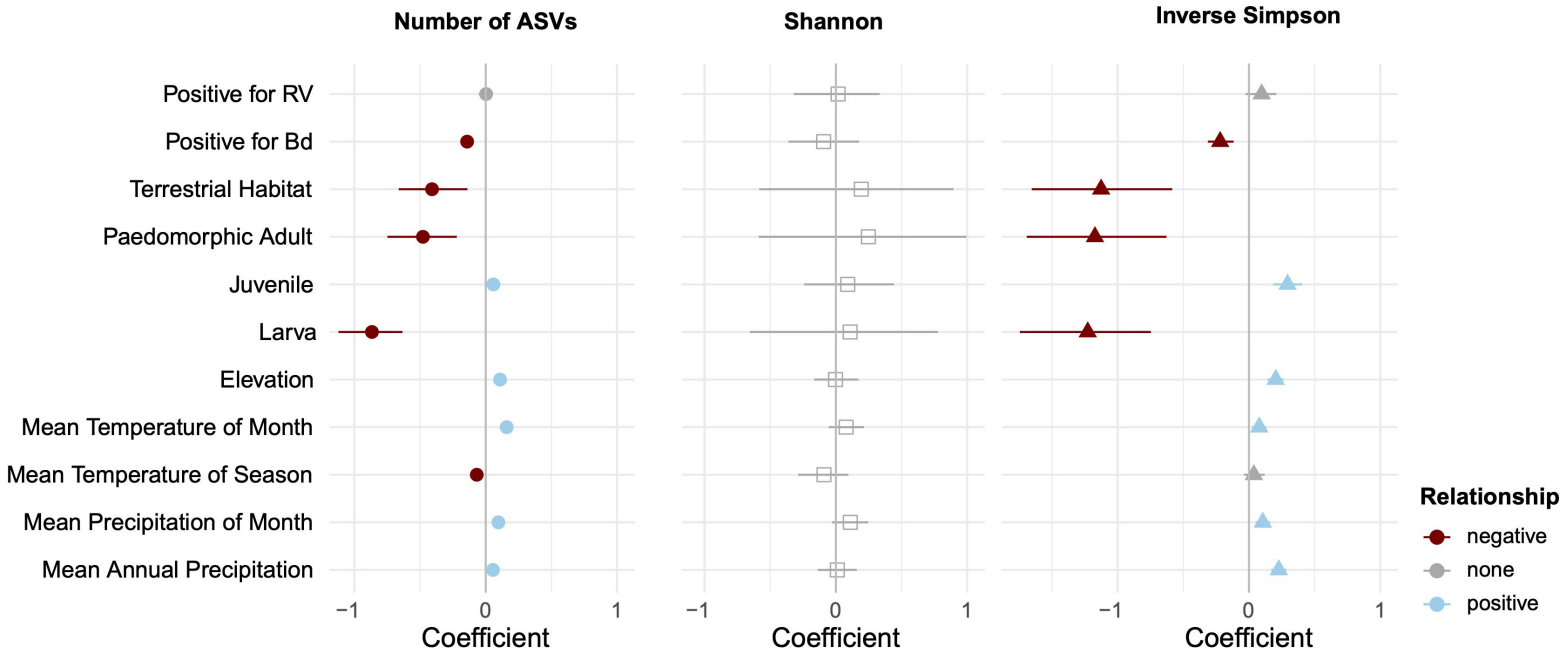
Relative contribution of explanatory variables to alpha diversity based on phylogenetic generalized linear mixed models. The figure contains posterior means and their 95% credible intervals. After accounting for phylogenetic non-independence, we found that ranavirus status has no impact on alpha diversity, but *Bd* presence has a slight negative impact. Warmer, wetter, and higher elevation sampling sites had slight positive effects on alpha diversity, but terrestrial habitats and some life history stages reduced microbial diversity.

Because differences among ecoregion may actually be the result of climatic differences among sites, as opposed to the biotic community (both of which are captured by ecoregion), we paired ecoregional comparisons with analyses directly using the underlying climatic data. When contrasting multiple predictor variables using CART analyses, environmental variables pertaining to temperature and precipitation either were not important or were secondary predictors of skin microbial diversity (**Figure 3**; **Supplementary Figures 5, 6**). However, when explicitly accounting for phylogeny in the PGLMMs, several abiotic variables had weak but significant positive relationships with alpha diversity: elevation, mean temperature of the month of collection, mean precipitation of season of collection, and mean precipitation of year (**Figure 4**).

#### 3.1.3 Relationship to ecological differences among hosts

Univariate comparisons of host ecology were limited to contrasts of salamander species that had samples of both aquatic and terrestrial individuals (*E. longicauda* and *E. tynerensis*). For these species, habitat was not a significant factor (χ^2^ < 0.76, *p* > 0.38 for both species across all alpha diversity metrics; **Supplementary Table 7**). When considered relative to other factors that partition variance in alpha diversity, CART analyses did not identify life stage or habitat as significant drivers of microbial diversity (**Figure 3**, **Supplementary Figures 5, 6**). However, when considered jointly with phylogeny and other explanatory factors in the PGLMM, a terrestrial ecology was negatively associated with alpha diversity for the observed ASVs and Inverse Simpson metrics, and skin microbiomes of both paedomorphic adult and larval plethodontids were associated with lower alpha diversity, while those of juvenile plethodontids had slightly increased alpha diversity (**Figure 4**).

### 3.2 Beta diversity

#### 3.2.1 Phylogenetic differences among hosts

**Figure 5:**
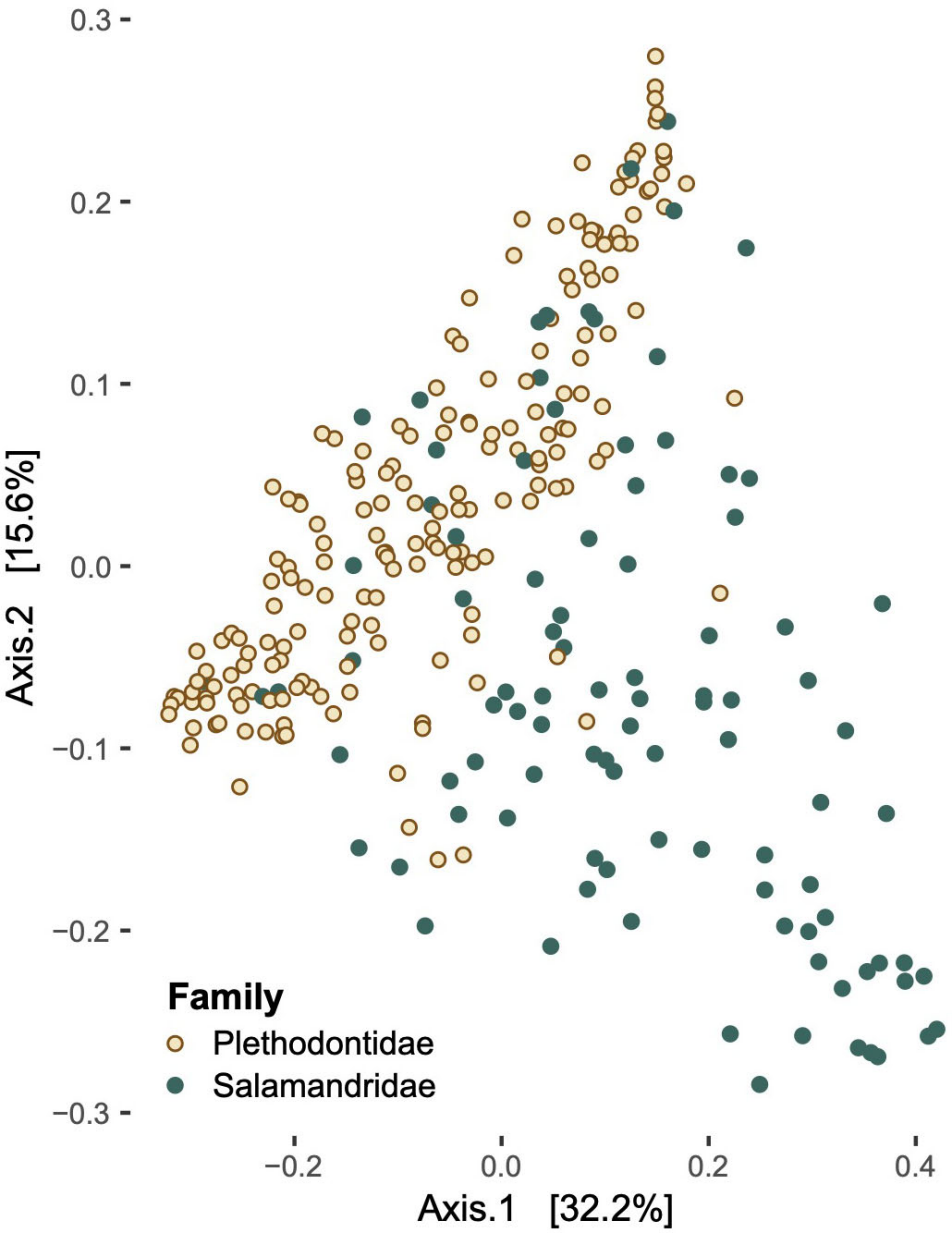
Principal coordinate analysis of beta diversity values in families Plethodontidae and Salamandridae.

Differences in microbial community composition (**Figure 5**), as measured by beta diversity, varied significantly in pairwise comparisons between salamander families, genera, and some species in both the weighted- and unweighted-UniFrac analyses (**Supplementary Table 7**). *Notophthalmus viridescens* exhibited distinctly different community composition when compared to each of the other salamander species in both analyses (*p* < 0.01). Similarly, microbial communities on *E. tynerensis* differed significantly from all other species (unweighted: *p* ≤ 0.03, r^2^ ≤ 0.05; weighted: *p* ≤ 0.045, r^2^ ≤ 0.05), except when compared to *P. angusticlavius* (*p* > 0.32, r^2^ ≤ 0.03 in both tests). Besides *E. tynerensis*, none of the other plethodontid microbiomes differed significantly between species (*p* > 0.1, r^2^ ≤ 0.05 in either metric; **Supplementary Table 7**).

#### 3.2.2 Environmental differences among hosts

To understand the role of the environment in shaping the composition of salamander microbiomes, we compared samples within species present in multiple ecoregions. For *N. viridescens*, all ecoregion comparisons were significantly different from each other with the exception of Arkansas Valley vs. Boston Mountains (*p* > 0.05, r^2 ^= 0.06) and Boston Mountains vs. Ozark Highlands (*p* = 0.21, r^2 ^= 0.02) in the unweighted-UniFrac comparisons (**Supplementary Table 8**). In the weighted-UniFrac, microbiomes from all ecoregions were significantly distinct, apart from the Arkansas Valley and Ozark Highlands comparison (p = 0.16, r^2 ^= 0.02). When comparing beta diversity across the three plethodontid species present in multiple ecoregions (*E. tynerensis*, *P. albagula*, and *P. angusticlavius*), there was no difference between the Boston Mountains and Ozark Highlands in any comparison (*p* > 0.1, r^2^ < 0.05; **Supplementary Table 9**).

We also used Mantel and partial Mantel tests to examine how beta diversity is shaped by climatic differences among sites. As results were similar with and without accounting for geographic distance (**Supplementary Tables 10, 11**), all following values thus refer to partial Mantel results. Overall, the mean temperature of the month had the strongest effect on beta diversity (all: r = 0.21; newts: r = 0.26; plethodontids: r = 0.17; *p* < 0.01 in each case); the mean temperature of the season and mean annual temperature had significant but weaker positive relationships with beta diversity (**Supplementary Table 10**). Elevation had a significant effect on beta diversity across all taxonomic groups but was not significant when considering salamander families separately after correcting for multiple comparisons (all: r = 0.12, *p* < 0.01; just newts: r = 0.14, *p* = 0.03; plethodontids: r = 0.11, *p* = 0.05). In newts, there was a positive relationship between mean precipitation during the month of capture and beta diversity (r = 0.30, *p* < 0.01), although no other climate variables influenced microbial community structure. In contrast, plethodontids had significant positive relationships with mean annual, seasonal, and monthly precipitation, with the latter being the strongest (annual: r = 0.11, *p* = 0.01; seasonal: r = 0.17, *p* < 0.01; monthly: r = 0.26, *p* < 0.01).

#### 3.2.3 Differences in beta diversity related to host ecology

Microbiomes from hosts found in aquatic and terrestrial habitats differed significantly in both unweighted- and weighted-UniFrac metrics (*p* < 0.01) when all samples were compared together. However, when testing only species that were sampled in both terrestrial and aquatic habitat types (*E. longicauda* and *E. tynerensis*), statistical significance differed based on whether the UniFrac test was weighted (unweighted: r^2 ^= 0.02, *p* = 0.02; weighted: r^2 ^= 0.02, *p* = 0.32; **Supplementary Table 9**).

To evaluate how life stage impacted beta diversity, we only examined plethodontids, as all newts in this study were adults (**Supplementary Table 9**). Juvenile plethodontids were significantly different from terrestrial adults, paedomorphic adults, and larvae in the unweighted UniFrac but did not differ from any other life stages in the weighted UniFrac. Adult plethodontids did not differ from paedomorphic adults but did differ from larvae in the unweighted UniFrac but not the weighted UniFrac. Paedomorphic adults did not differ in beta diversity from larvae (**Supplementary Table 9**).

### 3.3 Pathogen status and salamander microbiomes

#### 3.3.1 Presence of the fungal pathogen *B. dendrobatidis*

**Figure 6:**
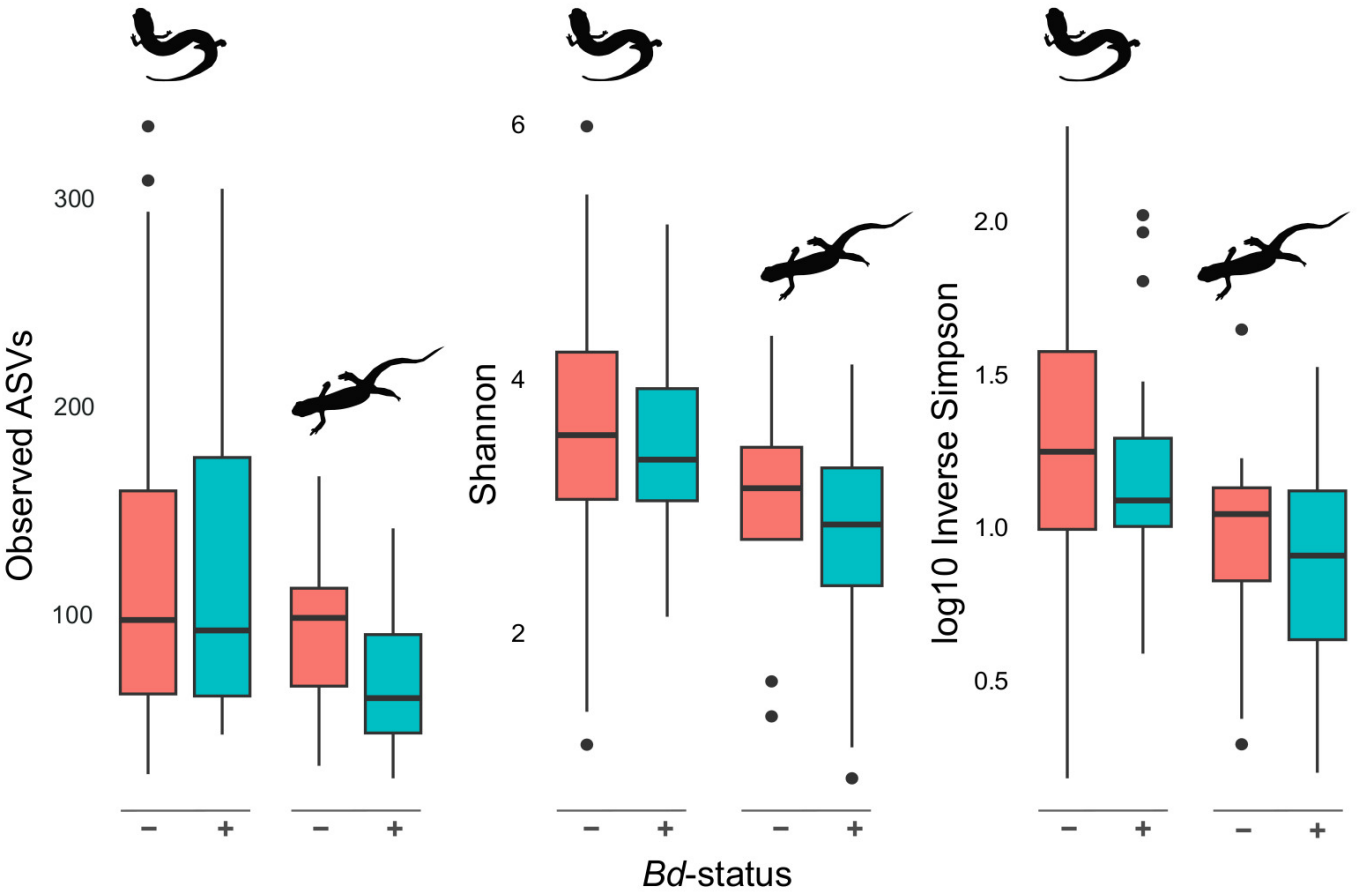
Alpha diversity differences based on *Bd* infection status. Alpha diversity of plethodontids and newts (left and right, respectively, within each set of plots) in the presence and absence of *Bd*. Overall, *Bd+* salamanders exhibit a slight but non-significant (**Supplementary Tables 4 & 5**) reduction in alpha diversity of the skin microbiome.

Animals without *Bd* present on their skin (*n* = 88) had skin microbial communities composed of Proteobacteria (59.30%), Firmicutes (12.99%), Bacteroidetes (13.92%), and Actinobacteria (5.40%). While microbial communities of *Bd*+ animals (n = 59) were composed of the same four groupings, they differed in terms of relative composition: Proteobacteria (81.62%), Firmicutes (6.64%), Bacteroidetes (6.47%), and Actinobacteria (3.06%). Two hundred fifty-nine of the 13,038 ASVs were identified to sequences in the antifungal isolates database. Of these, ten microbe species were differentially abundant between animals with *Bd* in the DESeq2 analysis and six were differentially abundant between animals without *Bd* in the ANCOM-BC analysis (**Supplementary Tables 12, 13**). When considering microbes without antifungal properties, we identified six differentially abundant microbes between animals with and without *Bd* present on the skin in the DESeq2 analysis and 23 in the ANCOM-BC analysis (**Supplementary Tables 14, 15**). However, most *Bd*+ animals were newts (*N. viridescens*; **Supplementary Data**). The presence of *Bd* covaried with family-level classifications; when separated by family, no univariate tests showed differences in alpha diversity by *Bd* status (**Supplementary Tables 5, 6**). In contrast, when accounting for phylogenetic dependence and other predictors with a PGLMM, the presence of *Bd* was associated with a slight decrease in alpha diversity in the observed ASVs, Shannon, and Inverse Simpson metrics (**Figures 4**, **6**).

#### 3.3.2 Presence of ranavirus

Ranavirus was not a significant predictor of alpha diversity, regardless of subdividing data among plethodontids or only newts (*p* = 1.0 for all univariate tests; **Supplementary Tables 5, 6**) and was not significant in either combined test of explanatory variables (**Figures 3**, **4**). Similarly, ranavirus status was not significant in either of the beta diversity analyses (r^2^ ≤ 0.01, *p* ≥ 0.34 for either metric; **Supplementary Tables 8, 9**).

## 4 Discussion

We set out to determine the relative importance of potential drivers of skin microbial diversity—phylogenetic distance, host ecology, host environment, and the presence of pathogens—in salamanders. Our results indicate that family-level differences (i.e., differences between Salamandridae and Plethodontidae) are consistently one of the strongest factors shaping the skin microbiomes at this taxonomic and geographic scale. Further analyses indicate these differences do not reflect a broader relationship with phylogenetic distance, but instead suggest that other differences that co-vary at the level of family may be influencing skin microbial diversity. Environmental and ecological factors play a secondary role in shaping both microbial (i.e., alpha) diversity and community differentiation (beta diversity), particularly among species. Although the presence of pathogens was not the most important factor determining microbiome structure or diversity, there was a slight, negative impact of *Bd* on the alpha diversity of salamander skin microbiomes. Together, these results provide further insight into the importance of multiple factors that simultaneously impact the skin microbiome in the presence of pathogens and underscore the importance of accounting for host family in microbiome studies.

### 4.1 Family-level differences significantly shape microbiomes

When we examined multiple drivers simultaneously, or accounted for their impacts by subdividing our data, the most important and consistent factor influencing salamander skin microbiomes was the family of the host, with differences in host ecology and climate becoming more important at the interspecific level. However, overall phylogenetic distance did a poor job of explaining these patterns, and most univariate comparisons among species or genera did not indicate increasing microbial dissimilarity with greater host differentiation. These results are generally similar to those of Buttimer et al. (2022), who recovered differences related to both host genera and habitat quality. In our study, family Salamandridae was distinct from Plethodontidae in both alpha and beta diversity metrics–with plethodontids having consistently higher alpha diversity than the newts–as well as overall microbial community membership (as indicated by beta diversity).

Differences in the skin microbiomes that correlate with phylogeny are sometimes attributed to phylosymbiosis, in which the microbes present in or on an organism are a result of long-term natural selection (Brucker and Bordenstein, 2012; Yeoh et al., 2017; Lim and Bordenstein, 2020). Families Plethodontidae and Salamandridae have been distinct for over 150 million years, and the genera *Eurycea* and *Plethodon* within Plethodontidae diverged around 75 million years ago (Zhang and Wake, 2009; Martin et al., 2016). This could provide sufficient evolutionary time for phylosymbiosis to develop, potentially driven by fitness consequences for animals that experience changes in the community composition of the skin microbiome (Becker et al., 2015; Bletz et al., 2018). However, while the strongest predictor of microbial abundance and diversity from most analyses in our study was host family (i.e., the greatest level of divergence in our dataset), we did not detect a strong phylogenetic signal at lower taxonomic levels. This suggests thatphylosymbiotic relationships are not the predominant driver of microbiome communities in these salamanders, or we are not able to identify them within our data at this phylogenetic scale. Differences in terms of the magnitude and sign of responses across phylogenetic scales are common (Graham et al., 2018), and are worth further consideration in this system with a broader phylogenetic and geographical dataset.

When comparing microbiomes within plethodontids, we did not find that genus was a significant predictor of microbiome composition, but we found instances where alpha or beta diversity of some host species differed significantly from others However, there was no clear pattern to the variation among hosts. For example, beta diversity of *E. tynerensis* differed significantly from all other salamander species in the unweighted-UniFrac test (**Supplementary Table 7**). In terms of alpha diversity, the CART analysis of Inverse Simpson values initially split *E. lucifuga* and *P. angusticlavius* from all other species. With no evidence for phylogenetic signals in host identity related to microbiomes, it is possible that unaccounted life history traits, behaviors, or diet are influencing these differences in microbial diversity. Microhabitat preferences also suggest one possibility linking the two species: adult *E. lucifuga* and *P. angusticlavius* both dwell in shallow caves and rock ledges (Petranka and Scott, 1998). Identifying and understanding these unaccounted drivers of among-species differences may allow us to better predict how changes in food or habitat availability may drive microbiome shifts in the future.

### 4.2 Differences in environment and host ecology

Although we identified environmental and ecological variables as secondary factors in structuring microbial communities in our study, they still played a significant role in explaining microbiome diversity. Although there were no differences by ecoregion, increases in average monthly precipitation, average temperature, and site elevation were associated with increases in alpha diversity. Factors relating to host ecology, such as habitat and life stage, were also significant in the PGLMMs, with juvenile life stage and terrestrial habitat exhibiting lower alpha diversity in comparison to other habitats and life stages. These results align with other studies, where host ecology and environment are important drivers of skin microbial diversity (e.g., Bletz et al., 2017a; Bird et al., 2018; Muletz Wolz et al., 2018; Smith et al., 2023), but these studies differ from one another in terms of the relative importance of predictors.

Although elevation, average precipitation, and average temperature of the month were secondary or tertiary drivers of microbial diversity, all had positive relationships with alpha diversity measures. When considered jointly with phylogeny, local climatic variables predicted alpha diversity; higher temperature or precipitation were associated with greater alpha diversity of microbial communities (**Figure 4**). None of these explanatory variables related to climate had a strong relationship to community membership alone, but after accounting for geographic distance among sites, partial Mantel tests indicated that microbial communities become more distinct between sites with different temperatures and those with different precipitation in each of the salamander families (**Supplementary Table 10**). We also compared the effect of ecoregion on the skin microbiome. Our analyses show that while there was no difference in microbial alpha diversity across the ecoregions, each ecoregion significantly differed in community composition from one or more other ecoregions in analyses of beta diversity. Our finding that abiotic factors are more important at the level of family are qualitatively similar to studies that focused on salamander species within the same family or genus (e.g., Kueneman et al., 2014; Walker et al., 2020), further supporting the idea that abiotic environmental variables become more important in structuring the microbiomes of closely related hosts.

When considering explanatory variables relating to host ecology and habitat, we expected to find significant differences in skin microbial diversity between terrestrial and aquatic environments, as well as between different life stages, similar to previous work across habitat types (Xu et al., 2020). The species in our study differ greatly in their life cycles. Adult newts, the only life stage of newts included herein, are aquatic (Petranka and Scott, 1998). In contrast, *Plethodon* spp. lay eggs on land and spend their entire lives as terrestrial animals; the genus *Eurycea* lays eggs in water, which hatch into aquatic larvae (Petranka and Scott, 1998). Larval *Eurycea* either transform into terrestrial juvenile forms which become terrestrial adults or remain in the water to become aquatic adults (Petranka and Scott, 1998). Despite these varied life histories, the difference in alpha diversity between terrestrial and aquatic environments was significant only in the PGLMM, which controls for phylogenetic dependence among hosts rather than directly assessing the effect of taxonomic categories. The difference in alpha diversity between life stages was also only evident in the PGLMM, in which being a juvenile plethodontid had a negative impact on the alpha diversity of the skin microbiome.We did find that juvenile plethodontids have significantly different beta diversity from larval, adult, and paedomorphic adult life stages in the unweighted-UniFrac analyses. Larval plethodontids were also significantly distinct from adults in the unweighted-UniFrac and from paedomorphic adults in the weighted-UniFrac; additionally, the two habitat types did have significantly different beta diversity in the unweighted-UniFrac analysis (**Supplementary Table 9**). The beta diversity of salamander microbiomes was not significantly different across different habitats when accounting for the phylogenetic distance and abundance among microbes (i.e., using the weighted UniFrac results; **Supplementary Table 9**). Overall, differences in beta diversity among life stage and habitat are less pronounced when accounting for abundance.

### 4.3 The presence of infection

We expected to find the presence of *Bd* on skin to be a substantial driver of the skin microbiome’s community composition, similar to previous work on *Anaxyrus boreas* toads and the closely related *Plethodon cinereus* salamanders (Kueneman et al., 2016; Muletz Wolz et al., 2019). After taking host phylogeny into account, we found that animals with *Bd* present on the skin showed slightly lower alpha diversity (**Figures 4**, **6**). Though only the PGLMM analyses provided statistical support for lower alpha diversity in the presence of disease (e.g., **Figure 4**), these results fit into our growing understanding of the interaction between chytridiomycosis and amphibian skin microbiomes (e.g., Harris et al., 2009; Bates et al., 2022).

Chytridiomycosis infects the skin of amphibians and disturbs osmoregulation and respiration in its host (Voyles et al., 2007; Rollins-Smith et al., 2011; Campbell et al., 2012; Martel et al., 2014, 2009). Recent studies have documented the significant prevalence of *Bd* and RV in North America, including among populations of amphibians in Oklahoma (Marhanka et al., 2017; Watters et al., 2018; Davis et al., 2019; Smith et al., 2019, 2019; Watters et al., 2021). Though none of our animals exhibited visible symptoms of disease (e.g., lethargy, increased skin shedding, and skin lesions; Martel et al., 2013, 2014; Voyles et al., 2007, 2009), the presence of *Bd* on the animal’s skin may indicate an ongoing or recent infection. Varying stages of infection have been shown to have very different impacts on amphibian skin microbiomes; while an overall decline in bacterial abundance has been observed related to *Bd* infections (Medina et al., 2017), this pattern involves the increase of some microbes and the decrease of others during and after infection (Jani and Briggs, 2014; Jani et al., 2021). Salamanders from our study may be at different stages of infection, which may minimize overall differences among “positive” animals if the microbiome’s alpha and beta diversity change during and again after infection, which could merit further study. Additionally, because we were not able to address changes in the function of the microbes present, there may be large differences in functional microbes but fewer differences in overall alpha or beta diversity (Jani et al., 2021).

Our results also indicate the presence of antifungal microbes in the skin microbiome (**Supplementary Table 13**), a feature that has been identified in multiple studies (Becker et al., 2015; Woodhams et al., 2015). Laboratory manipulation of antifungal microbes on amphibians’ skin in has been linked to improved chytridiomycosis survival rates (Harris et al., 2009; Bletz et al., 2013; Kueneman et al., 2016; Bletz et al., 2018), which suggests beneficial microbes may naturally increase in abundance during infection in animals that tolerate chytridiomycosis (Campbell et al., 2019). Although there were no significant differences in alpha or beta diversity of antifungal microbes between animals with *Bd* status in our study, there were multiple differentially abundant antifungal microbe species when contrasting animals with and without *Bd* (**Supplementary Tables 12, 13**). However, microbes with antifungal properties may decline in number during infection, suggesting a more nuanced relationship between microbial function and microbiome composition (Medina et al., 2017). More studies about *Bd* and its effect on the amphibian skin microbiome are needed to aid in answering these questions.

In the case of RV infections, we expected possible virus-driven variation in the composition of the skin microbiome, based on previous work that indicated amphibians with ranavirus infection exhibit changes in microbial community composition (Campbell et al., 2019). However, as infection with RV takes place internally, it is therefore possible that the presence of RV is less likely to influence the skin microbiome than *Bd* (Gray and Chinchar, 2015). In our study, there was not a significant relationship between ranavirus presence and alpha or beta diversity in any tests. This does not necessarily indicate that there is no relationship between ranavirus infection and changes in the skin microbiome. A recent study has found that the composition of the gut microbiome of *Rana sylvatica* may influence stress response to ranaviral infection (Hughey et al., 2023). Future studies focused on symptomatic animals or gut microbiomes may provide greater insights into the host-ranavirus-microbiome relationship.

## 5 Conclusions

Effective conservation planning requires an understanding of not only a target species, but also the impact of its interactions with other species, including microbes (Hird, 2017). This study provides a snapshot of the skin microbiomes of six salamander species and how these microbiomes vary across different habitats, varying environments, and phylogenetic distances. While we found that the most important determinant of skin microbiome composition is the family of the host, the research herein suggests that the skin microbiomes are not more divergent as phylogenetic distance among hosts increases (as would be expected under phylosymbiosis theory). After family-level differences are accounted for, host ecology, host environment, and the presence of disease all influence the diversity and community composition of microbiomes. Furthermore, the presence of differentially abundant microbes linked to antifungal properties on the skin of animals testing positive for *Bd* suggests a possible interaction occurring between pathogens and skin microbiomes. As infectious diseases continue to decimate amphibian populations, characterizing host microbiomes and examining the function of antifungal microbes is more important than ever.

## Data availability statement

The datasets presented in this study can be found in online repositories. The names of the repository/repositories and accession number(s) can be found below: https://www.ncbi.nlm.nih.gov/, PRJNA1002333.

## Ethics statement

The animal study was approved by University of Oklahoma Institutional Animal Care and Use Committee. The study was conducted in accordance with the local legislation and institutional requirements.

## Author contributions

MK: Conceptualization, Data curation, Formal analysis, Investigation, Methodology, Software, Visualization, Writing – original draft, Writing – review & editing. SS: Investigation, Validation, Writing – review & editing. DB: Methodology, Software, Supervision, Writing – review & editing, Validation. JW: Writing – review & editing, Data curation, Funding acquisition, Investigation, Project administration. KM: Funding acquisition, Supervision, Writing – review & editing, Validation. CS: Funding acquisition, Validation, Writing – review & editing, Conceptualization, Investigation, Methodology, Project administration, Resources, Supervision. HL: Conceptualization, Methodology, Supervision, Validation, Writing – review & editing, Formal analysis, Visualization, Writing – original draft.
