# Supplementary material for: Family shapes microbiome differences in Oklahoma salamanders
Source: Front Microbiomes. 2024 Mar 13;3:1277645. doi: 10.3389/frmbi.2024.1277645 (PMC12993562; doi:10.3389/frmbi.2024.1277645)
Supplement: Supplementary file 1 [file DataSheet_1.docx]

***Supplementary Material***

# Supplementary Data

All code and formatted data can be accessed at: https://github.com/mkirsch44/16S_Salamander_Microbial_Pipeline

**Supplementary Data:** Collection and microbial diversity data for each sample. Information includes the OMNH voucher ID, the Oklahoma Department of Wildlife Conservation (ODWC) Field ID, the total ASVs, the observed ASVs, and disease status for both ranavirus (RV) and *Batrachochytrium dendrobatidis* (*Bd*) (Y = present; N = not present; NA = not screened). Disease data from Davis et al., 2019; Marhanka et al., 2017; and Watters et al., 2018.

| ***OMNH*** ***ID*** | ***ODWC Field ID*** | ***Family*** | ***Genus*** | ***Species*** | ***Ecoregion*** | ***Habitat*** | ***Life Stage*** | ***Total ASVs*** | ***Observed ASVs*** | ***RV*** | ***Bd*** | ***Year*** |
| --- | --- | --- | --- | --- | --- | --- | --- | --- | --- | --- | --- | --- |
| 43983 | 277 | Salamandridae | *Notophthalmus* | *viridescens* | Ouachita Mountains | aquatic | adult | 3777 | 54 | N | N | 2015 |
| 43984 | 278 | Salamandridae | *Notophthalmus* | *viridescens* | Ouachita Mountains | aquatic | adult | 5371 | 62 | N | Y | 2015 |
| 43986 | 281 | Salamandridae | *Notophthalmus* | *viridescens* | Ouachita Mountains | aquatic | adult | 705 | 54 | N | Y | 2015 |
| 43987 | 282 | Salamandridae | *Notophthalmus* | *viridescens* | Ouachita Mountains | aquatic | adult | 4149 | 44 | N | N | 2015 |
| 43991 | 288 | Salamandridae | *Notophthalmus* | *viridescens* | Ouachita Mountains | aquatic | adult | 1758 | 44 | N | NA | 2015 |
| 43935 | 329 | Plethodontidae | *Eurycea* | *lucifuga* | Ozark Highlands | terrestrial | adult | 5881 | 452 | N | N | 2015 |
| 43948 | 341 | Plethodontidae | *Plethodon* | *albagula* | Ozark Highlands | terrestrial | juvenile | 491 | 45 | Y | N | 2015 |
| 43936 | 346 | Plethodontidae | *Eurycea* | *lucifuga* | Ozark Highlands | terrestrial | adult | 2283 | 46 | Y | N | 2015 |
| 43972 | 350 | Plethodontidae | *Plethodon* | *angusticlavius* | Ozark Highlands | terrestrial | juvenile | 774 | 44 | N | N | 2015 |
| 43952 | 351 | Plethodontidae | *Plethodon* | *albagula* | Ozark Highlands | terrestrial | adult | 12380 | 94 | Y | N | 2015 |
| 43953 | 355 | Plethodontidae | *Plethodon* | *albagula* | Ozark Highlands | terrestrial | juvenile | 1772 | 155 | N | N | 2015 |
| 43954 | 356 | Plethodontidae | *Plethodon* | *albagula* | Ozark Highlands | terrestrial | juvenile | 1297 | 90 | Y | N | 2015 |
| 43955 | 357 | Plethodontidae | *Plethodon* | *albagula* | Ozark Highlands | terrestrial | juvenile | 742 | 38 | Y | N | 2015 |
| 43944 | 358 | Plethodontidae | *Eurycea* | *tynerensis* | Ozark Highlands | terrestrial | adult | 6838 | 463 | Y | Y | 2015 |
| 43956 | 359 | Plethodontidae | *Plethodon* | *albagula* | Ozark Highlands | terrestrial | juvenile | 320 | 13 | Y | N | 2015 |
| 43957 | 361 | Plethodontidae | *Plethodon* | *albagula* | Ozark Highlands | terrestrial | adult | 64965 | 663 | N | N | 2015 |
| 43903 | 378 | Plethodontidae | *Eurycea* | *longicauda* | Ozark Highlands | terrestrial | adult | 1425 | 48 | N | Y | 2015 |
| 43945 | 399 | Plethodontidae | *Eurycea* | *tynerensis* | Ozark Highlands | aquatic | paedomorphic adult | 12820 | 609 | N | Y | 2015 |
| 43908 | 402 | Plethodontidae | *Eurycea* | *longicauda* | Ozark Highlands | terrestrial | adult | 1163 | 38 | NA | NA | 2015 |
| 43909 | 403 | Plethodontidae | *Eurycea* | *longicauda* | Ozark Highlands | terrestrial | adult | 2091 | 63 | N | Y | 2015 |
| 43910 | 404 | Plethodontidae | *Eurycea* | *longicauda* | Ozark Highlands | terrestrial | adult | 5413 | 263 | N | N | 2015 |
| 43911 | 405 | Plethodontidae | *Eurycea* | *longicauda* | Ozark Highlands | terrestrial | adult | 605 | 45 | N | N | 2015 |
| 43912 | 407 | Plethodontidae | *Eurycea* | *longicauda* | Ozark Highlands | terrestrial | adult | 1092 | 54 | N | Y | 2015 |
| 43913 | 408 | Plethodontidae | *Eurycea* | *longicauda* | Ozark Highlands | terrestrial | adult | 3976 | 168 | NA | NA | 2015 |
| 43914 | 409 | Plethodontidae | *Eurycea* | *longicauda* | Ozark Highlands | terrestrial | adult | 1302 | 56 | NA | NA | 2015 |
| 44758 | 412 | Plethodontidae | *Eurycea* | *tynerensis* | Ozark Highlands | terrestrial | adult | 745 | 46 | Y | N | 2015 |
| 44750 | 413 | Plethodontidae | *Eurycea* | *longicauda* | Ozark Highlands | aquatic | larvae | 1205 | 87 | N | Y | 2015 |
| 44753 | 414 | Plethodontidae | *Eurycea* | *lucifuga* | Ozark Highlands | terrestrial | larvae | 8474 | 127 | N | N | 2015 |
| 43976 | 416 | Plethodontidae | *Plethodon* | *angusticlavius* | Ozark Highlands | terrestrial | juvenile | 583 | 37 | N | N | 2015 |
| 44759 | 417 | Plethodontidae | *Eurycea* | *tynerensis* | Ozark Highlands | terrestrial | adult | 1322 | 59 | NA | NA | 2015 |
| 44751 | 418 | Plethodontidae | *Eurycea* | *longicauda* | Ozark Highlands | aquatic | larvae | 10979 | 239 | NA | NA | 2015 |
| 43916 | 421 | Plethodontidae | *Eurycea* | *longicauda* | Ozark Highlands | terrestrial | juvenile | 5064 | 449 | N | Y | 2015 |
| 43917 | 423 | Plethodontidae | *Eurycea* | *longicauda* | Ozark Highlands | terrestrial | adult | 2372 | 147 | NA | NA | 2015 |
| 43962 | 427 | Plethodontidae | *Plethodon* | *albagula* | Ozark Highlands | terrestrial | adult | 518 | 46 | Y | N | 2015 |
| 43938 | 428 | Plethodontidae | *Eurycea* | *lucifuga* | Ozark Highlands | terrestrial | adult | 2179 | 197 | Y | N | 2015 |
| 43920 | 441 | Plethodontidae | *Eurycea* | *longicauda* | Ozark Highlands | terrestrial | adult | 201 | 17 | Y | N | 2015 |
| 43921 | 442 | Plethodontidae | *Eurycea* | *longicauda* | Ozark Highlands | terrestrial | adult | 230 | 19 | Y | N | 2015 |
| 43922 | 443 | Plethodontidae | *Eurycea* | *longicauda* | Ozark Highlands | terrestrial | adult | 149 | 12 | Y | N | 2015 |
| 43923 | 444 | Plethodontidae | *Eurycea* | *longicauda* | Ozark Highlands | terrestrial | juvenile | 146 | 12 | Y | Y | 2015 |
| 43963 | 449 | Plethodontidae | *Plethodon* | *albagula* | Ozark Highlands | terrestrial | adult | 73 | 12 | N | Y | 2015 |
| 43964 | 450 | Plethodontidae | *Plethodon* | *albagula* | Ozark Highlands | terrestrial | adult | 111 | 12 | NA | NA | 2015 |
| 43965 | 451 | Plethodontidae | *Plethodon* | *albagula* | Ozark Highlands | terrestrial | juvenile | 350 | 45 | N | N | 2015 |
| 43966 | 452 | Plethodontidae | *Plethodon* | *albagula* | Ozark Highlands | terrestrial | adult | 227 | 33 | N | N | 2015 |
| 43967 | 453 | Plethodontidae | *Plethodon* | *albagula* | Ozark Highlands | terrestrial | adult | 237 | 28 | N | Y | 2015 |
| 43968 | 454 | Plethodontidae | *Plethodon* | *albagula* | Ozark Highlands | terrestrial | adult | 137 | 49 | N | N | 2015 |
| 43977 | 455 | Plethodontidae | *Plethodon* | *angusticlavius* | Ozark Highlands | terrestrial | juvenile | 204 | 6 | N | N | 2015 |
| 43978 | 456 | Plethodontidae | *Plethodon* | *angusticlavius* | Ozark Highlands | terrestrial | juvenile | 129 | 4 | N | Y | 2015 |
| 43928 | 457 | Plethodontidae | *Eurycea* | *longicauda* | Ozark Highlands | terrestrial | adult | 193 | 3 | N | N | 2015 |
| 43929 | 458 | Plethodontidae | *Eurycea* | *longicauda* | Ozark Highlands | terrestrial | adult | 92 | 3 | N | N | 2015 |
| 43930 | 459 | Plethodontidae | *Eurycea* | *longicauda* | Ozark Highlands | terrestrial | juvenile | 175 | 7 | NA | NA | 2015 |
| 43931 | 460 | Plethodontidae | *Eurycea* | *longicauda* | Ozark Highlands | terrestrial | juvenile | 79 | 9 | N | Y | 2015 |
| 43969 | 476 | Plethodontidae | *Plethodon* | *albagula* | Ozark Highlands | terrestrial | adult | 52 | 7 | N | N | 2015 |
| 43970 | 480 | Plethodontidae | *Plethodon* | *albagula* | Ozark Highlands | terrestrial | juvenile | 6684 | 518 | Y | N | 2015 |
| 43996 | 481 | Salamandridae | *Notophthalmus* | *viridescens* | Ozark Highlands | aquatic | adult | 277 | 24 | N | Y | 2015 |
| 43997 | 482 | Salamandridae | *Notophthalmus* | *viridescens* | Ozark Highlands | aquatic | adult | 459 | 21 | N | Y | 2015 |
| 44000 | 485 | Salamandridae | *Notophthalmus* | *viridescens* | Ozark Highlands | aquatic | adult | 2500 | 37 | N | Y | 2015 |
| 44001 | 486 | Salamandridae | *Notophthalmus* | *viridescens* | Ozark Highlands | aquatic | adult | 1086 | 48 | NA | NA | 2015 |
| 44002 | 487 | Salamandridae | *Notophthalmus* | *viridescens* | Ozark Highlands | aquatic | adult | 532 | 34 | N | Y | 2015 |
| 44003 | 488 | Salamandridae | *Notophthalmus* | *viridescens* | Ozark Highlands | aquatic | adult | 1322 | 61 | N | Y | 2015 |
| 43946 | 520 | Plethodontidae | *Eurycea* | *tynerensis* | Ozark Highlands | aquatic | paedomorphic adult | 2893 | 99 | N | N | 2015 |
| 44023 | 538 | Salamandridae | *Notophthalmus* | *viridescens* | Ozark Highlands | aquatic | adult | 1201 | 33 | N | Y | 2015 |
| 44024 | 539 | Salamandridae | *Notophthalmus* | *viridescens* | Ozark Highlands | aquatic | adult | 427 | 12 | N | Y | 2015 |
| 44025 | 540 | Salamandridae | *Notophthalmus* | *viridescens* | Ozark Highlands | aquatic | adult | 194 | 20 | NA | NA | 2015 |
| 44026 | 541 | Salamandridae | *Notophthalmus* | *viridescens* | Ozark Highlands | aquatic | adult | 233 | 14 | NA | NA | 2015 |
| 44027 | 542 | Salamandridae | *Notophthalmus* | *viridescens* | Ozark Highlands | aquatic | adult | 318 | 24 | NA | NA | 2015 |
| 44028 | 543 | Salamandridae | *Notophthalmus* | *viridescens* | Ozark Highlands | aquatic | adult | 493 | 24 | NA | NA | 2015 |
| 43971 | 560 | Plethodontidae | *Plethodon* | *albagula* | Ozark Highlands | terrestrial | juvenile | 2268 | 153 | Y | Y | 2015 |
| 43932 | 590 | Plethodontidae | *Eurycea* | *longicauda* | Ozark Highlands | terrestrial | adult | 400 | 33 | N | Y | 2015 |
| 43934 | 592 | Plethodontidae | *Eurycea* | *longicauda* | Ozark Highlands | terrestrial | adult | 9673 | 256 | N | N | 2015 |
| 44031 | 627 | Salamandridae | *Notophthalmus* | *viridescens* | Arkansas Valley | aquatic | adult | 1480 | 87 | Y | Y | 2015 |
| 44032 | 628 | Salamandridae | *Notophthalmus* | *viridescens* | Arkansas Valley | aquatic | adult | 2979 | 133 | N | Y | 2015 |
| 44033 | 629 | Salamandridae | *Notophthalmus* | *viridescens* | Arkansas Valley | aquatic | adult | 12074 | 111 | Y | N | 2015 |
| 44034 | 630 | Salamandridae | *Notophthalmus* | *viridescens* | Arkansas Valley | aquatic | adult | 4042 | 181 | Y | Y | 2015 |
| 44042 | 633 | Salamandridae | *Notophthalmus* | *viridescens* | Arkansas Valley | aquatic | adult | 6642 | 99 | Y | Y | 2015 |
| 44047 | 638 | Salamandridae | *Notophthalmus* | *viridescens* | Arkansas Valley | aquatic | adult | 690 | 29 | N | NA | 2015 |
| 44050 | 641 | Salamandridae | *Notophthalmus* | *viridescens* | Arkansas Valley | aquatic | adult | 1289 | 55 | N | NA | 2015 |
| 44051 | 642 | Salamandridae | *Notophthalmus* | *viridescens* | Arkansas Valley | aquatic | adult | 22818 | 134 | Y | NA | 2015 |
| 44037 | 683 | Salamandridae | *Notophthalmus* | *viridescens* | Arkansas Valley | aquatic | adult | 863 | 39 | N | Y | 2015 |
| 44038 | 684 | Salamandridae | *Notophthalmus* | *viridescens* | Arkansas Valley | aquatic | adult | 20940 | 95 | Y | Y | 2015 |
| 44039 | 685 | Salamandridae | *Notophthalmus* | *viridescens* | Arkansas Valley | aquatic | adult | 39218 | 127 | N | Y | 2015 |
| 44040 | 692 | Salamandridae | *Notophthalmus* | *viridescens* | Arkansas Valley | aquatic | adult | 4395 | 101 | Y | Y | 2015 |
| 44041 | 693 | Salamandridae | *Notophthalmus* | *viridescens* | Arkansas Valley | aquatic | adult | 2010 | 77 | N | Y | 2015 |
| 44070 | 1007 | Salamandridae | *Notophthalmus* | *viridescens* | Ouachita Mountains | aquatic | adult | 25960 | 146 | Y | Y | 2015 |
| 44071 | 1008 | Salamandridae | *Notophthalmus* | *viridescens* | Ouachita Mountains | aquatic | adult | 41991 | 292 | Y | Y | 2015 |
| 44073 | 1040 | Salamandridae | *Notophthalmus* | *viridescens* | Ouachita Mountains | aquatic | adult | 50618 | 386 | N | N | 2015 |
| 44074 | 1041 | Salamandridae | *Notophthalmus* | *viridescens* | Ouachita Mountains | aquatic | adult | 13153 | 181 | Y | Y | 2015 |
| 44075 | 1042 | Salamandridae | *Notophthalmus* | *viridescens* | Ouachita Mountains | aquatic | adult | 429627 | 703 | N | NA | 2015 |
| 44076 | 1043 | Salamandridae | *Notophthalmus* | *viridescens* | Ouachita Mountains | aquatic | adult | 53121 | 284 | N | N | 2015 |
| 44085 | 1052 | Salamandridae | *Notophthalmus* | *viridescens* | Ouachita Mountains | aquatic | adult | 34547 | 243 | N | Y | 2015 |
| 44056 | 1136 | Salamandridae | *Notophthalmus* | *viridescens* | Ouachita Mountains | aquatic | adult | 15470 | 244 | N | Y | 2015 |
| 44057 | 1137 | Salamandridae | *Notophthalmus* | *viridescens* | Ouachita Mountains | aquatic | adult | 9580 | 185 | N | Y | 2015 |
| 44059 | 1176 | Salamandridae | *Notophthalmus* | *viridescens* | Ouachita Mountains | aquatic | adult | 10639 | 223 | N | Y | 2015 |
| 44060 | 1177 | Salamandridae | *Notophthalmus* | *viridescens* | Ouachita Mountains | aquatic | adult | 24368 | 308 | N | Y | 2015 |
| 44062 | 1179 | Salamandridae | *Notophthalmus* | *viridescens* | Ouachita Mountains | aquatic | adult | 99506 | 246 | N | Y | 2015 |
| 46000 | 1570 | Plethodontidae | *Eurycea* | *tynerensis* | Boston Mountains | terrestrial | adult | 2549 | 79 | NA | NA | 2016 |
| 46001 | 1573 | Plethodontidae | *Eurycea* | *tynerensis* | Boston Mountains | terrestrial | adult | 357 | 42 | N | N | 2016 |
| 46002 | 1574 | Plethodontidae | *Eurycea* | *tynerensis* | Boston Mountains | aquatic | paedomorphic adult | 488 | 15 | N | N | 2016 |
| 46003 | 1575 | Plethodontidae | *Eurycea* | *tynerensis* | Boston Mountains | aquatic | paedomorphic adult | 870 | 10 | N | N | 2016 |
| 46004 | 1576 | Plethodontidae | *Eurycea* | *tynerensis* | Boston Mountains | aquatic | paedomorphic adult | 946 | 19 | N | N | 2016 |
| 46005 | 1577 | Plethodontidae | *Eurycea* | *tynerensis* | Boston Mountains | terrestrial | adult | 1170 | 26 | N | N | 2016 |
| 46006 | 1578 | Plethodontidae | *Eurycea* | *tynerensis* | Boston Mountains | terrestrial | adult | 3330 | 81 | N | Y | 2016 |
| 46007 | 1579 | Plethodontidae | *Eurycea* | *tynerensis* | Boston Mountains | aquatic | paedomorphic adult | 1867 | 43 | N | N | 2016 |
| 46008 | 1580 | Plethodontidae | *Eurycea* | *tynerensis* | Boston Mountains | aquatic | paedomorphic adult | 15099 | 70 | N | N | 2016 |
| 46009 | 1581 | Plethodontidae | *Eurycea* | *tynerensis* | Boston Mountains | aquatic | paedomorphic adult | 870 | 26 | N | N | 2016 |
| 46010 | 1582 | Plethodontidae | *Eurycea* | *tynerensis* | Boston Mountains | aquatic | paedomorphic adult | 1273 | 26 | N | N | 2016 |
| 46011 | 1583 | Plethodontidae | *Eurycea* | *tynerensis* | Boston Mountains | terrestrial | adult | 2809 | 97 | N | N | 2016 |
| 46095 | 1590 | Salamandridae | *Notophthalmus* | *viridescens* | Boston Mountains | aquatic | adult | 2663 | 46 | N | Y | 2016 |
| 46096 | 1597 | Salamandridae | *Notophthalmus* | *viridescens* | Boston Mountains | aquatic | adult | 865 | 23 | N | Y | 2016 |
| 46097 | 1598 | Salamandridae | *Notophthalmus* | *viridescens* | Boston Mountains | aquatic | adult | 37877 | 77 | N | Y | 2016 |
| 46098 | 1599 | Salamandridae | *Notophthalmus* | *viridescens* | Boston Mountains | aquatic | adult | 6924 | 88 | N | Y | 2016 |
| 46099 | 1600 | Salamandridae | *Notophthalmus* | *viridescens* | Boston Mountains | aquatic | adult | 639 | 14 | N | Y | 2016 |
| 46100 | 1601 | Salamandridae | *Notophthalmus* | *viridescens* | Boston Mountains | aquatic | adult | 7147 | 97 | N | Y | 2016 |
| 46101 | 1602 | Salamandridae | *Notophthalmus* | *viridescens* | Boston Mountains | aquatic | adult | 22773 | 110 | N | Y | 2016 |
| 46102 | 1603 | Salamandridae | *Notophthalmus* | *viridescens* | Boston Mountains | aquatic | adult | 4575 | 82 | N | Y | 2016 |
| 46103 | 1604 | Salamandridae | *Notophthalmus* | *viridescens* | Boston Mountains | aquatic | adult | 516 | 22 | N | Y | 2016 |
| 46104 | 1615 | Salamandridae | *Notophthalmus* | *viridescens* | Boston Mountains | aquatic | adult | 5298 | 66 | N | Y | 2016 |
| 45983 | 1639 | Plethodontidae | *Eurycea* | *lucifuga* | Ozark Highlands | terrestrial | adult | 309744 | 4062 | N | N | 2016 |
| 46206 | 1640 | Plethodontidae | *Eurycea* | *tynerensis* | Ozark Highlands | aquatic | larvae | 1670 | 54 | N | N | 2016 |
| 46012 | 1641 | Plethodontidae | *Eurycea* | *tynerensis* | Ozark Highlands | aquatic | larvae | 10736 | 146 | N | N | 2016 |
| 46013 | 1642 | Plethodontidae | *Eurycea* | *tynerensis* | Ozark Highlands | terrestrial | juvenile | 20732 | 227 | N | N | 2016 |
| 46014 | 1643 | Plethodontidae | *Eurycea* | *tynerensis* | Ozark Highlands | aquatic | paedomorphic adult | 20467 | 203 | N | Y | 2016 |
| 46015 | 1644 | Plethodontidae | *Eurycea* | *tynerensis* | Ozark Highlands | aquatic | larvae | 4722 | 127 | N | N | 2016 |
| 46016 | 1645 | Plethodontidae | *Eurycea* | *tynerensis* | Ozark Highlands | aquatic | paedomorphic adult | 12983 | 214 | N | N | 2016 |
| 46017 | 1646 | Plethodontidae | *Eurycea* | *tynerensis* | Ozark Highlands | aquatic | paedomorphic adult | 13815 | 171 | N | N | 2016 |
| 46050 | 1650 | Salamandridae | *Notophthalmus* | *viridescens* | Ozark Highlands | aquatic | adult | 11 | 10 | N | Y | 2016 |
| 46051 | 1651 | Salamandridae | *Notophthalmus* | *viridescens* | Ozark Highlands | aquatic | adult | 844 | 24 | N | Y | 2016 |
| 46052 | 1652 | Salamandridae | *Notophthalmus* | *viridescens* | Ozark Highlands | aquatic | adult | 1025 | 32 | Y | Y | 2016 |
| 46053 | 1653 | Salamandridae | *Notophthalmus* | *viridescens* | Ozark Highlands | aquatic | adult | 4000 | 70 | N | Y | 2016 |
| 46054 | 1654 | Salamandridae | *Notophthalmus* | *viridescens* | Ozark Highlands | aquatic | adult | 9886 | 87 | N | Y | 2016 |
| 46055 | 1655 | Salamandridae | *Notophthalmus* | *viridescens* | Ozark Highlands | aquatic | adult | 2221 | 47 | Y | Y | 2016 |
| 46056 | 1656 | Salamandridae | *Notophthalmus* | *viridescens* | Ozark Highlands | aquatic | adult | 9474 | 87 | N | Y | 2016 |
| 46057 | 1657 | Salamandridae | *Notophthalmus* | *viridescens* | Ozark Highlands | aquatic | adult | 7027 | 100 | N | Y | 2016 |
| 46058 | 1658 | Salamandridae | *Notophthalmus* | *viridescens* | Ozark Highlands | aquatic | adult | 3621 | 66 | N | Y | 2016 |
| 46059 | 1659 | Salamandridae | *Notophthalmus* | *viridescens* | Ozark Highlands | aquatic | adult | 2378 | 51 | Y | Y | 2016 |
| 46060 | 1660 | Salamandridae | *Notophthalmus* | *viridescens* | Ozark Highlands | aquatic | adult | 11025 | 49 | NA | NA | 2016 |
| 46061 | 1661 | Salamandridae | *Notophthalmus* | *viridescens* | Ozark Highlands | aquatic | adult | 84 | 16 | NA | NA | 2016 |
| 46062 | 1662 | Salamandridae | *Notophthalmus* | *viridescens* | Ozark Highlands | aquatic | adult | 384 | 15 | NA | NA | 2016 |
| 46063 | 1663 | Salamandridae | *Notophthalmus* | *viridescens* | Ozark Highlands | aquatic | adult | 1446 | 63 | NA | NA | 2016 |
| 46064 | 1664 | Salamandridae | *Notophthalmus* | *viridescens* | Ozark Highlands | aquatic | adult | 7548 | 95 | NA | NA | 2016 |
| 46065 | 1665 | Salamandridae | *Notophthalmus* | *viridescens* | Ozark Highlands | aquatic | adult | 285 | 16 | NA | NA | 2016 |
| 46066 | 1666 | Salamandridae | *Notophthalmus* | *viridescens* | Ozark Highlands | aquatic | adult | 1413 | 34 | NA | NA | 2016 |
| 46067 | 1667 | Salamandridae | *Notophthalmus* | *viridescens* | Ozark Highlands | aquatic | adult | 498 | 32 | NA | NA | 2016 |
| 46068 | 1668 | Salamandridae | *Notophthalmus* | *viridescens* | Ozark Highlands | aquatic | adult | 656 | 40 | NA | NA | 2016 |
| 46069 | 1669 | Salamandridae | *Notophthalmus* | *viridescens* | Ozark Highlands | aquatic | adult | 190 | 7 | NA | NA | 2016 |
| 46070 | 1670 | Salamandridae | *Notophthalmus* | *viridescens* | Ozark Highlands | aquatic | adult | 1705 | 53 | NA | NA | 2016 |
| 46071 | 1671 | Salamandridae | *Notophthalmus* | *viridescens* | Ozark Highlands | aquatic | adult | 1187 | 45 | NA | NA | 2016 |
| 46072 | 1672 | Salamandridae | *Notophthalmus* | *viridescens* | Ozark Highlands | aquatic | adult | 5662 | 112 | NA | NA | 2016 |
| 46073 | 1673 | Salamandridae | *Notophthalmus* | *viridescens* | Ozark Highlands | aquatic | adult | 1103 | 38 | NA | NA | 2016 |
| 46074 | 1674 | Salamandridae | *Notophthalmus* | *viridescens* | Ozark Highlands | aquatic | adult | 23063 | 74 | NA | NA | 2016 |
| 46075 | 1675 | Salamandridae | *Notophthalmus* | *viridescens* | Ozark Highlands | aquatic | adult | 2239 | 39 | NA | NA | 2016 |
| 46076 | 1676 | Salamandridae | *Notophthalmus* | *viridescens* | Ozark Highlands | aquatic | adult | 2423 | 50 | NA | NA | 2016 |
| 46077 | 1677 | Salamandridae | *Notophthalmus* | *viridescens* | Ozark Highlands | aquatic | adult | 10326 | 114 | NA | NA | 2016 |
| 46078 | 1686 | Salamandridae | *Notophthalmus* | *viridescens* | Ozark Highlands | aquatic | adult | 10832 | 64 | NA | NA | 2016 |
| 46079 | 1687 | Salamandridae | *Notophthalmus* | *viridescens* | Ozark Highlands | aquatic | adult | 1885 | 59 | NA | NA | 2016 |
| 46080 | 1688 | Salamandridae | *Notophthalmus* | *viridescens* | Ozark Highlands | aquatic | adult | 1424 | 56 | NA | NA | 2016 |
| 46081 | 1689 | Salamandridae | *Notophthalmus* | *viridescens* | Ozark Highlands | aquatic | adult | 2811 | 56 | NA | NA | 2016 |
| 46082 | 1690 | Salamandridae | *Notophthalmus* | *viridescens* | Ozark Highlands | aquatic | adult | 686 | 43 | NA | NA | 2016 |
| 46018 | 1692 | Plethodontidae | *Eurycea* | *tynerensis* | Ozark Highlands | aquatic | larvae | 2142 | 89 | N | N | 2016 |
| 46019 | 1693 | Plethodontidae | *Eurycea* | *tynerensis* | Ozark Highlands | aquatic | larvae | 924 | 56 | N | N | 2016 |
| 46020 | 1695 | Plethodontidae | *Eurycea* | *tynerensis* | Ozark Highlands | aquatic | larvae | 539 | 66 | N | N | 2016 |
| 46021 | 1696 | Plethodontidae | *Eurycea* | *tynerensis* | Ozark Highlands | aquatic | larvae | 639 | 120 | NA | NA | 2016 |
| 46022 | 1697 | Plethodontidae | *Eurycea* | *tynerensis* | Ozark Highlands | aquatic | larvae | 1717 | 61 | N | N | 2016 |
| 46025 | 1698 | Plethodontidae | *Plethodon* | *albagula* | Boston Mountains | terrestrial | juvenile | 15152 | 180 | N | Y | 2016 |
| 46041 | 1707 | Plethodontidae | *Plethodon* | *angusticlavius* | Boston Mountains | terrestrial | juvenile | 239 | 20 | N | N | 2016 |
| 46042 | 1708 | Plethodontidae | *Plethodon* | *angusticlavius* | Boston Mountains | terrestrial | juvenile | 1524 | 231 | N | N | 2016 |
| 46043 | 1709 | Plethodontidae | *Plethodon* | *angusticlavius* | Boston Mountains | terrestrial | juvenile | 3449 | 424 | N | N | 2016 |
| 46044 | 1712 | Plethodontidae | *Plethodon* | *angusticlavius* | Boston Mountains | terrestrial | juvenile | 6836 | 155 | NA | NA | 2016 |
| 45984 | 1728 | Plethodontidae | *Eurycea* | *lucifuga* | Ozark Highlands | terrestrial | adult | 183 | 18 | N | N | 2016 |
| 45986 | 1730 | Plethodontidae | *Eurycea* | *lucifuga* | Ozark Highlands | terrestrial | juvenile | 3643 | 242 | N | N | 2016 |
| 46027 | 1732 | Plethodontidae | *Plethodon* | *albagula* | Ozark Highlands | terrestrial | juvenile | 631 | 28 | N | N | 2016 |
| 46028 | 1733 | Plethodontidae | *Plethodon* | *albagula* | Ozark Highlands | terrestrial | juvenile | 3448 | 60 | N | N | 2016 |
| 45987 | 1736 | Plethodontidae | *Eurycea* | *lucifuga* | Ozark Highlands | terrestrial | juvenile | 830 | 64 | N | N | 2016 |
| 45988 | 1737 | Plethodontidae | *Eurycea* | *lucifuga* | Ozark Highlands | terrestrial | adult | 880 | 72 | N | N | 2016 |
| 45989 | 1741 | Plethodontidae | *Eurycea* | *lucifuga* | Ozark Highlands | terrestrial | adult | 11585 | 152 | N | N | 2016 |
| 46023 | 1745 | Plethodontidae | *Eurycea* | *tynerensis* | Ozark Highlands | aquatic | paedomorphic adult | 11834 | 111 | N | Y | 2016 |
| 46029 | 1751 | Plethodontidae | *Plethodon* | *albagula* | Ozark Highlands | terrestrial | juvenile | 39164 | 787 | N | N | 2016 |
| 46024 | 1755 | Plethodontidae | *Eurycea* | *tynerensis* | Ozark Highlands | aquatic | larvae | 6185 | 193 | N | N | 2016 |
| 46045 | 1763 | Plethodontidae | *Plethodon* | *angusticlavius* | Boston Mountains | terrestrial | juvenile | 2 | 2 | N | N | 2016 |
| 46046 | 1764 | Plethodontidae | *Plethodon* | *angusticlavius* | Boston Mountains | terrestrial | juvenile | 6 | 4 | N | N | 2016 |
| 46047 | 1765 | Plethodontidae | *Plethodon* | *angusticlavius* | Boston Mountains | terrestrial | juvenile | 4 | 3 | N | N | 2016 |
| 46048 | 1766 | Plethodontidae | *Plethodon* | *angusticlavius* | Boston Mountains | terrestrial | adult | 1 | 1 | N | N | 2016 |
| 46049 | 1767 | Plethodontidae | *Plethodon* | *angusticlavius* | Boston Mountains | terrestrial | adult | 13 | 8 | N | N | 2016 |
| 46026 | 1768 | Plethodontidae | *Plethodon* | *albagula* | Boston Mountains | terrestrial | juvenile | 17 | 11 | N | N | 2016 |
| 46083 | 1774 | Salamandridae | *Notophthalmus* | *viridescens* | Ozark Highlands | aquatic | adult | 10 | 6 | NA | NA | 2016 |
| 45990 | 1818 | Plethodontidae | *Eurycea* | *lucifuga* | Ozark Highlands | terrestrial | adult | 46 | 29 | N | N | 2016 |
| 45991 | 1819 | Plethodontidae | *Eurycea* | *lucifuga* | Ozark Highlands | terrestrial | adult | 3 | 3 | N | N | 2016 |
| 45992 | 1825 | Plethodontidae | *Eurycea* | *lucifuga* | Ozark Highlands | terrestrial | adult | 9 | 7 | N | N | 2016 |
| 45993 | 1826 | Plethodontidae | *Eurycea* | *lucifuga* | Ozark Highlands | terrestrial | adult | 9 | 9 | N | Y | 2016 |
| 46040 | 1828 | Plethodontidae | *Plethodon* | *albagula* | Ozark Highlands | terrestrial | juvenile | 202 | 28 | NA | NA | 2016 |
| 46030 | 2230 | Plethodontidae | *Plethodon* | *albagula* | Ozark Highlands | terrestrial | juvenile | 58978 | 2776 | NA | NA | 2016 |
| 46031 | 2231 | Plethodontidae | *Plethodon* | *albagula* | Ozark Highlands | terrestrial | juvenile | 4607 | 55 | N | N | 2016 |
| 46032 | 2232 | Plethodontidae | *Plethodon* | *albagula* | Ozark Highlands | terrestrial | juvenile | 13346 | 318 | N | N | 2016 |
| 46033 | 2233 | Plethodontidae | *Plethodon* | *albagula* | Ozark Highlands | terrestrial | adult | 2802 | 83 | N | N | 2016 |
| 46034 | 2234 | Plethodontidae | *Plethodon* | *albagula* | Ozark Highlands | terrestrial | juvenile | 8678 | 179 | N | N | 2016 |
| 46035 | 2235 | Plethodontidae | *Plethodon* | *albagula* | Ozark Highlands | terrestrial | juvenile | 103626 | 388 | N | N | 2016 |
| 45994 | 2236 | Plethodontidae | *Eurycea* | *lucifuga* | Ozark Highlands | terrestrial | adult | 68964 | 582 | Y | N | 2016 |
| 45995 | 2237 | Plethodontidae | *Eurycea* | *lucifuga* | Ozark Highlands | terrestrial | adult | 51476 | 200 | N | N | 2016 |
| 45996 | 2238 | Plethodontidae | *Eurycea* | *lucifuga* | Ozark Highlands | terrestrial | adult | 61090 | 411 | N | N | 2016 |
| 45997 | 2239 | Plethodontidae | *Eurycea* | *lucifuga* | Ozark Highlands | terrestrial | adult | 14306 | 142 | N | Y | 2016 |
| 45998 | 2240 | Plethodontidae | *Eurycea* | *lucifuga* | Ozark Highlands | terrestrial | adult | 74216 | 267 | N | N | 2016 |
| 45977 | 2258 | Plethodontidae | *Eurycea* | *longicauda* | Ozark Highlands | terrestrial | juvenile | 12129 | 149 | N | Y | 2016 |
| 46084 | 2265 | Salamandridae | *Notophthalmus* | *viridescens* | Ozark Highlands | aquatic | adult | 18540 | 256 | N | Y | 2016 |
| 46085 | 2266 | Salamandridae | *Notophthalmus* | *viridescens* | Ozark Highlands | aquatic | adult | 567 | 9 | N | N | 2016 |
| 46086 | 2267 | Salamandridae | *Notophthalmus* | *viridescens* | Ozark Highlands | aquatic | adult | 6477 | 111 | N | Y | 2016 |
| 45978 | 2277 | Plethodontidae | *Eurycea* | *longicauda* | Ozark Highlands | terrestrial | adult | 13851 | 602 | N | N | 2016 |
| 45979 | 2278 | Plethodontidae | *Eurycea* | *longicauda* | Ozark Highlands | terrestrial | adult | 6532 | 214 | N | N | 2016 |
| 45980 | 2279 | Plethodontidae | *Eurycea* | *longicauda* | Ozark Highlands | terrestrial | juvenile | 24715 | 574 | N | Y | 2016 |
| 46036 | 2296 | Plethodontidae | *Plethodon* | *albagula* | Ozark Highlands | terrestrial | juvenile | 2254 | 125 | N | N | 2016 |
| 46037 | 2297 | Plethodontidae | *Plethodon* | *albagula* | Ozark Highlands | terrestrial | juvenile | 3112 | 84 | N | N | 2016 |
| 46038 | 2298 | Plethodontidae | *Plethodon* | *albagula* | Ozark Highlands | terrestrial | juvenile | 7458 | 331 | N | N | 2016 |
| 46039 | 2299 | Plethodontidae | *Plethodon* | *albagula* | Ozark Highlands | terrestrial | juvenile | 1589 | 45 | N | N | 2016 |
| 45982 | 2300 | Plethodontidae | *Eurycea* | *longicauda* | Ozark Highlands | terrestrial | adult | 15186 | 545 | N | N | 2016 |
| 46087 | 2301 | Salamandridae | *Notophthalmus* | *viridescens* | Ozark Highlands | aquatic | adult | 18131 | 173 | N | N | 2016 |
| 46088 | 2302 | Salamandridae | *Notophthalmus* | *viridescens* | Ozark Highlands | aquatic | adult | 6494 | 128 | N | Y | 2016 |
| 46089 | 2303 | Salamandridae | *Notophthalmus* | *viridescens* | Ozark Highlands | aquatic | adult | 11035 | 151 | N | Y | 2016 |
| 46092 | 2306 | Salamandridae | *Notophthalmus* | *viridescens* | Ozark Highlands | aquatic | adult | 98639 | 374 | N | N | 2016 |
| 46093 | 2307 | Salamandridae | *Notophthalmus* | *viridescens* | Ozark Highlands | aquatic | adult | 10907 | 157 | N | N | 2016 |
| 46094 | 2309 | Salamandridae | *Notophthalmus* | *viridescens* | Ozark Highlands | aquatic | adult | 3301 | 79 | NA | NA | 2016 |
| 47329 | 2994 | Salamandridae | *Notophthalmus* | *viridescens* | Ouachita Mountains | aquatic | adult | 2347 | 37 | NA | NA | NA |
| 47906 | 3540 | Plethodontidae | *Eurycea* | *longicauda* | Ozark Highlands | terrestrial | juvenile | 1289 | 56 | N | N | 2018 |
| 47885 | 3541 | Plethodontidae | *Eurycea* | *tynerensis* | Ozark Highlands | terrestrial | adult | 45222 | 509 | N | N | 2018 |
| 47886 | 3542 | Plethodontidae | *Eurycea* | *tynerensis* | Ozark Highlands | terrestrial | adult | 13283 | 362 | N | N | 2018 |
| 47887 | 3547 | Plethodontidae | *Eurycea* | *tynerensis* | Ozark Highlands | aquatic | paedomorphic adult | 54351 | 478 | N | Y | 2018 |
| 47888 | 3548 | Plethodontidae | *Eurycea* | *tynerensis* | Ozark Highlands | aquatic | paedomorphic adult | 57314 | 300 | N | N | 2018 |
| 47889 | 3549 | Plethodontidae | *Eurycea* | *tynerensis* | Ozark Highlands | aquatic | paedomorphic adult | 66865 | 280 | N | Y | 2018 |
| 47890 | 3550 | Plethodontidae | *Eurycea* | *tynerensis* | Ozark Highlands | terrestrial | adult | 7449 | 176 | N | N | 2018 |
| 47891 | 3551 | Plethodontidae | *Eurycea* | *tynerensis* | Ozark Highlands | terrestrial | adult | 1129 | 88 | N | N | 2018 |
| 47892 | 3552 | Plethodontidae | *Eurycea* | *tynerensis* | Ozark Highlands | aquatic | larvae | 2146 | 149 | N | N | 2018 |
| 47872 | 3556 | Plethodontidae | *Eurycea* | *tynerensis* | Ozark Highlands | terrestrial | adult | 1024 | 86 | N | N | 2018 |
| 47873 | 3557 | Plethodontidae | *Eurycea* | *tynerensis* | Ozark Highlands | terrestrial | adult | 2217 | 104 | N | N | 2018 |
| 47893 | 3561 | Plethodontidae | *Eurycea* | *tynerensis* | Ozark Highlands | terrestrial | adult | 283 | 37 | N | Y | 2018 |
| 47902 | 3565 | Plethodontidae | *Plethodon* | *angusticlavius* | Ozark Highlands | terrestrial | adult | 455 | 41 | N | N | 2018 |
| 47903 | 3566 | Plethodontidae | *Plethodon* | *angusticlavius* | Ozark Highlands | terrestrial | adult | 9974 | 643 | N | N | 2018 |
| 47904 | 3567 | Plethodontidae | *Plethodon* | *angusticlavius* | Ozark Highlands | terrestrial | adult | 2404 | 150 | N | N | 2018 |
| 47905 | 3568 | Plethodontidae | *Plethodon* | *angusticlavius* | Ozark Highlands | terrestrial | adult | 1788 | 68 | N | N | 2018 |
| 47898 | 3569 | Plethodontidae | *Plethodon* | *albagula* | Ozark Highlands | terrestrial | adult | 502 | 31 | N | N | 2018 |
| 47899 | 3570 | Plethodontidae | *Plethodon* | *albagula* | Ozark Highlands | terrestrial | adult | 315 | 19 | N | N | 2018 |
| 47900 | 3571 | Plethodontidae | *Plethodon* | *albagula* | Ozark Highlands | terrestrial | adult | 1112 | 67 | N | N | 2018 |
| 47901 | 3572 | Plethodontidae | *Plethodon* | *albagula* | Ozark Highlands | terrestrial | juvenile | 90292 | 487 | N | N | 2018 |
| 47883 | 3573 | Plethodontidae | *Eurycea* | *lucifuga* | Ozark Highlands | terrestrial | adult | 1409 | 75 | N | N | 2018 |
| 47884 | 3574 | Plethodontidae | *Eurycea* | *lucifuga* | Ozark Highlands | terrestrial | adult | 61565 | 670 | N | N | 2018 |
| 47896 | 3580 | Plethodontidae | *Eurycea* | *tynerensis* | Ozark Highlands | terrestrial | adult | 5997 | 226 | N | N | 2018 |
| 47874 | 3582 | Plethodontidae | *Eurycea* | *longicauda* | Ozark Highlands | terrestrial | adult | 7523 | 337 | N | N | 2018 |
| 47875 | 3583 | Plethodontidae | *Eurycea* | *longicauda* | Ozark Highlands | terrestrial | adult | 14635 | 377 | N | N | 2018 |
| 47876 | 3584 | Plethodontidae | *Eurycea* | *longicauda* | Ozark Highlands | terrestrial | adult | 5561 | 85 | N | N | 2018 |
| 47877 | 3585 | Plethodontidae | *Eurycea* | *longicauda* | Ozark Highlands | terrestrial | adult | 2293 | 99 | N | N | 2018 |
| 47878 | 3586 | Plethodontidae | *Eurycea* | *longicauda* | Ozark Highlands | terrestrial | adult | 7900 | 177 | N | N | 2018 |
| 47879 | 3587 | Plethodontidae | *Eurycea* | *longicauda* | Ozark Highlands | terrestrial | adult | 69581 | 529 | N | N | 2018 |
| 47880 | 3588 | Plethodontidae | *Eurycea* | *longicauda* | Ozark Highlands | terrestrial | adult | 6772 | 162 | N | N | 2018 |
| 47881 | 3589 | Plethodontidae | *Eurycea* | *longicauda* | Ozark Highlands | terrestrial | adult | 4851 | 340 | N | N | 2018 |
| 47882 | 3590 | Plethodontidae | *Eurycea* | *longicauda* | Ozark Highlands | terrestrial | juvenile | 2476 | 60 | N | N | 2018 |
| 47897 | 3594 | Plethodontidae | *Eurycea* | *tynerensis* | Ozark Highlands | terrestrial | adult | 69532 | 523 | N | N | 2018 |
| 48318 | 4173 | Plethodontidae | *Eurycea* | *lucifuga* | Ozark Highlands | terrestrial | juvenile | 3 | 2 | N | N | 2021 |
| *N/A* | 4177 | Plethodontidae | *Eurycea* | *lucifuga* | Ozark Highlands | terrestrial | adult or juvenile | 1 | 1 | N | N | 2021 |
| 48322 | 4178 | Plethodontidae | *Eurycea* | *tynerensis* | Ozark Highlands | terrestrial | adult | 316 | 15 | N | Y | 2021 |
| 48323 | 4179 | Plethodontidae | *Eurycea* | *tynerensis* | Ozark Highlands | terrestrial | adult | 482 | 10 | N | N | 2021 |
| *N/A* | 4180 | Plethodontidae | *Eurycea* | *tynerensis* | Ozark Highlands | terrestrial | adult or juvenile | 183 | 9 | N | Y | 2021 |
| *N/A* | 4181 | Plethodontidae | *Eurycea* | *tynerensis* | Ozark Highlands | terrestrial | adult or juvenile | 4524 | 213 | N | Y | 2021 |
| *N/A* | 4194 | Plethodontidae | *Eurycea* | *tynerensis* | Ozark Highlands | terrestrial | adult or juvenile | 727 | 56 | N | N | 2021 |
| *N/A* | 4196 | Plethodontidae | *Eurycea* | *tynerensis* | Ozark Highlands | terrestrial | adult or juvenile | 351 | 8 | N | N | 2021 |
| *N/A* | 4197 | Plethodontidae | *Eurycea* | *tynerensis* | Ozark Highlands | terrestrial | adult or juvenile | 5323 | 122 | N | N | 2021 |
| *N/A* | 4198 | Plethodontidae | *Eurycea* | *tynerensis* | Ozark Highlands | terrestrial | adult or juvenile | 1112 | 62 | N | Y | 2021 |
| *N/A* | 4199 | Plethodontidae | *Eurycea* | *tynerensis* | Ozark Highlands | terrestrial | adult or juvenile | 13 | 12 | N | N | 2021 |
| *N/A* | 4202 | Plethodontidae | *Eurycea* | *tynerensis* | Ozark Highlands | terrestrial | adult or juvenile | 478 | 25 | N | N | 2021 |
| *N/A* | 4203 | Plethodontidae | *Eurycea* | *tynerensis* | Ozark Highlands | terrestrial | adult or juvenile | 161 | 38 | N | N | 2021 |
| *N/A* | 4205 | Plethodontidae | *Eurycea* | *tynerensis* | Ozark Highlands | terrestrial | adult or juvenile | 12478 | 71 | N | N | 2021 |
| *N/A* | 4207 | Plethodontidae | *Eurycea* | *tynerensis* | Ozark Highlands | terrestrial | adult or juvenile | 152 | 22 | N | N | 2021 |
| *N/A* | 4208 | Plethodontidae | *Eurycea* | *tynerensis* | Ozark Highlands | terrestrial | adult or juvenile | 201 | 23 | N | N | 2021 |
| *N/A* | 4209 | Plethodontidae | *Eurycea* | *tynerensis* | Ozark Highlands | terrestrial | adult or juvenile | 186 | 33 | N | N | 2021 |
| *N/A* | 4210 | Plethodontidae | *Eurycea* | *tynerensis* | Ozark Highlands | terrestrial | adult or juvenile | 700 | 45 | N | N | 2021 |
| *N/A* | 4273 | Plethodontidae | *Eurycea* | *tynerensis* | Ozark Highlands | aquatic | larvae | 515 | 28 | N | N | 2021 |
| 48325 | 4274 | Plethodontidae | *Eurycea* | *tynerensis* | Ozark Highlands | aquatic | larvae | 276 | 20 | N | N | 2021 |
| 48326 | 4275 | Plethodontidae | *Eurycea* | *tynerensis* | Ozark Highlands | aquatic | larvae | 54591 | 169 | N | N | 2021 |
| *N/A* | 4276 | Plethodontidae | *Eurycea* | *tynerensis* | Ozark Highlands | aquatic | larvae | 239 | 13 | N | N | 2021 |
| 48327 | 4277 | Plethodontidae | *Plethodon* | *albagula* | Ozark Highlands | terrestrial | juvenile | 173 | 17 | N | N | 2021 |
| 48316 | 4278 | Plethodontidae | *Eurycea* | *longicauda* | Ozark Highlands | terrestrial | adult | 168 | 17 | N | N | 2021 |
| 48319 | 4281 | Plethodontidae | *Eurycea* | *lucifuga* | Ozark Highlands | terrestrial | adult | 903 | 31 | N | N | 2021 |
| *N/A* | 4289 | Plethodontidae | *Plethodon* | *albagula* | Ozark Highlands | terrestrial | adult or juvenile | 347 | 12 | N | N | 2021 |
| *N/A* | 4290 | Plethodontidae | *Eurycea* | *longicauda* | Ozark Highlands | terrestrial | adult or juvenile | 489 | 30 | N | N | 2021 |
| *N/A* | 4291 | Plethodontidae | *Eurycea* | *longicauda* | Ozark Highlands | terrestrial | adult or juvenile | 1597 | 94 | N | N | 2021 |

# Supplementary Figures and Tables

## Supplementary Figures

**Supplementary Figure 1.** Rarefaction curves showing the number of observed taxonomic units (top) and Shannon diversity (bottom) based upon the sequencing depth.

**
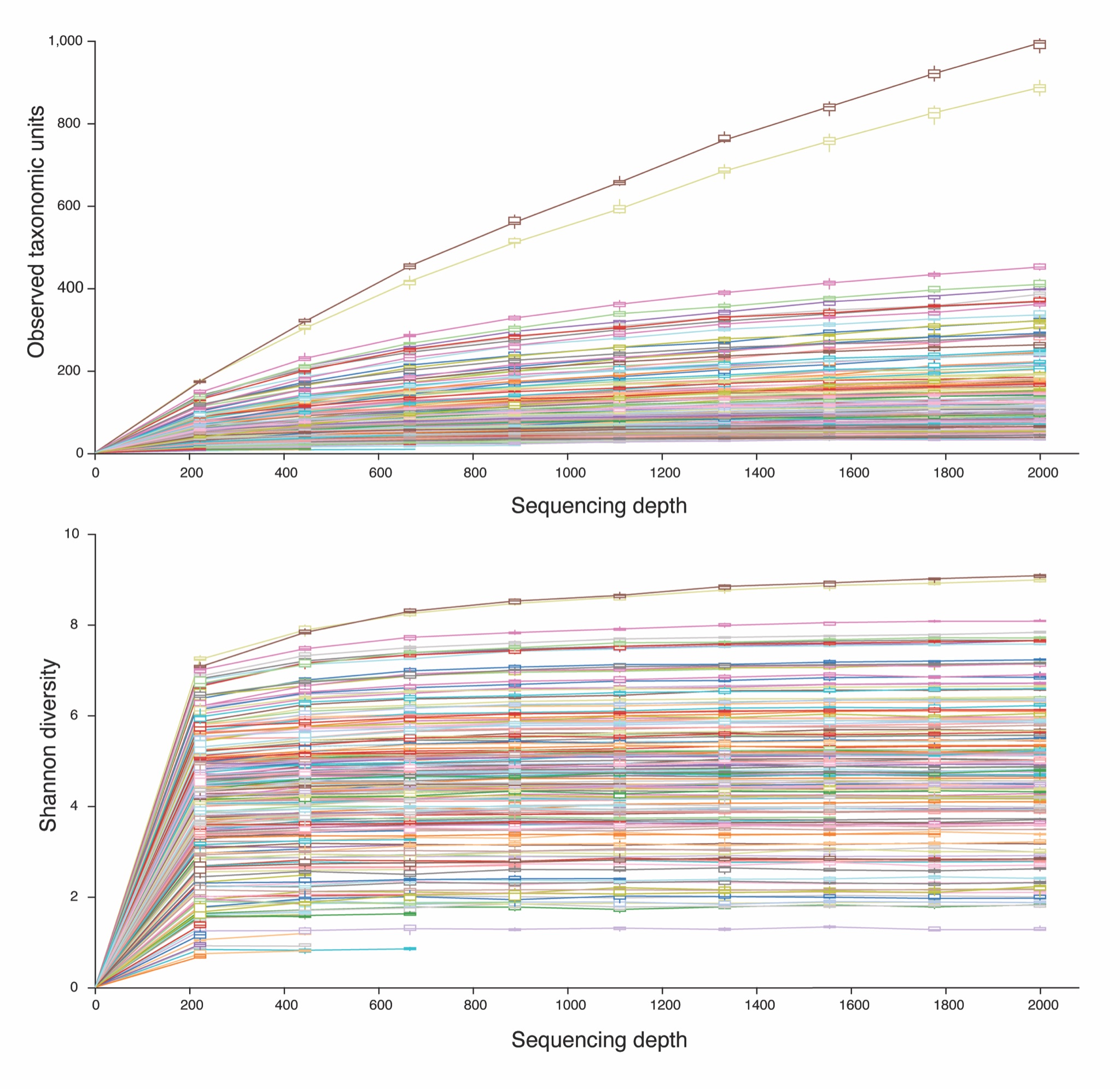
**

**Supplementary Figure 2.** Relative proportions of microbial families present in the skin microbiomes of salamanders across all six species.


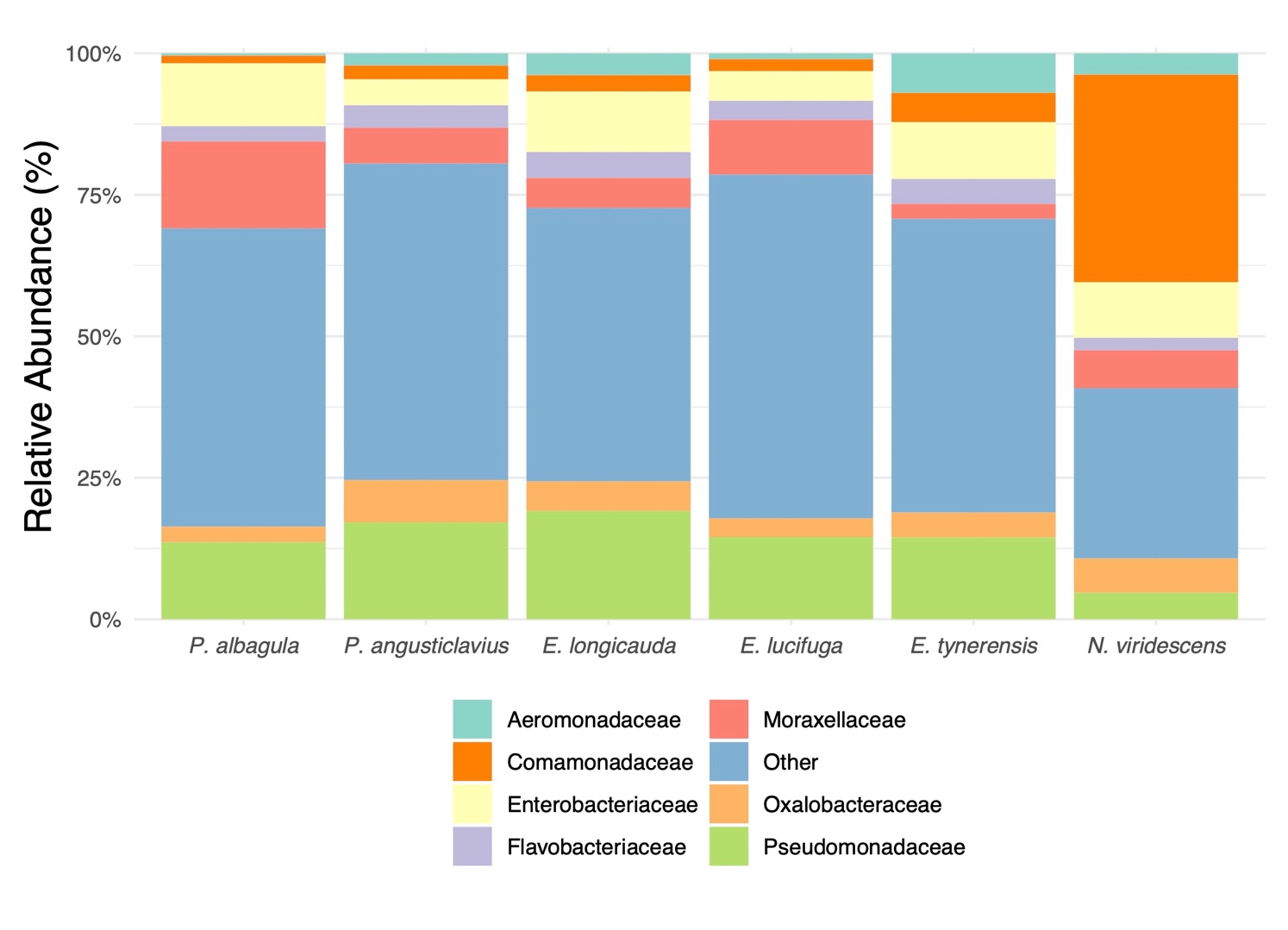


**Supplementary Figure 3:** Taxonomy stacked barplot of relative proportions of microbial phyla among host species, relative to habitat (H) and life stage (LS), both shown on the right of the plot.


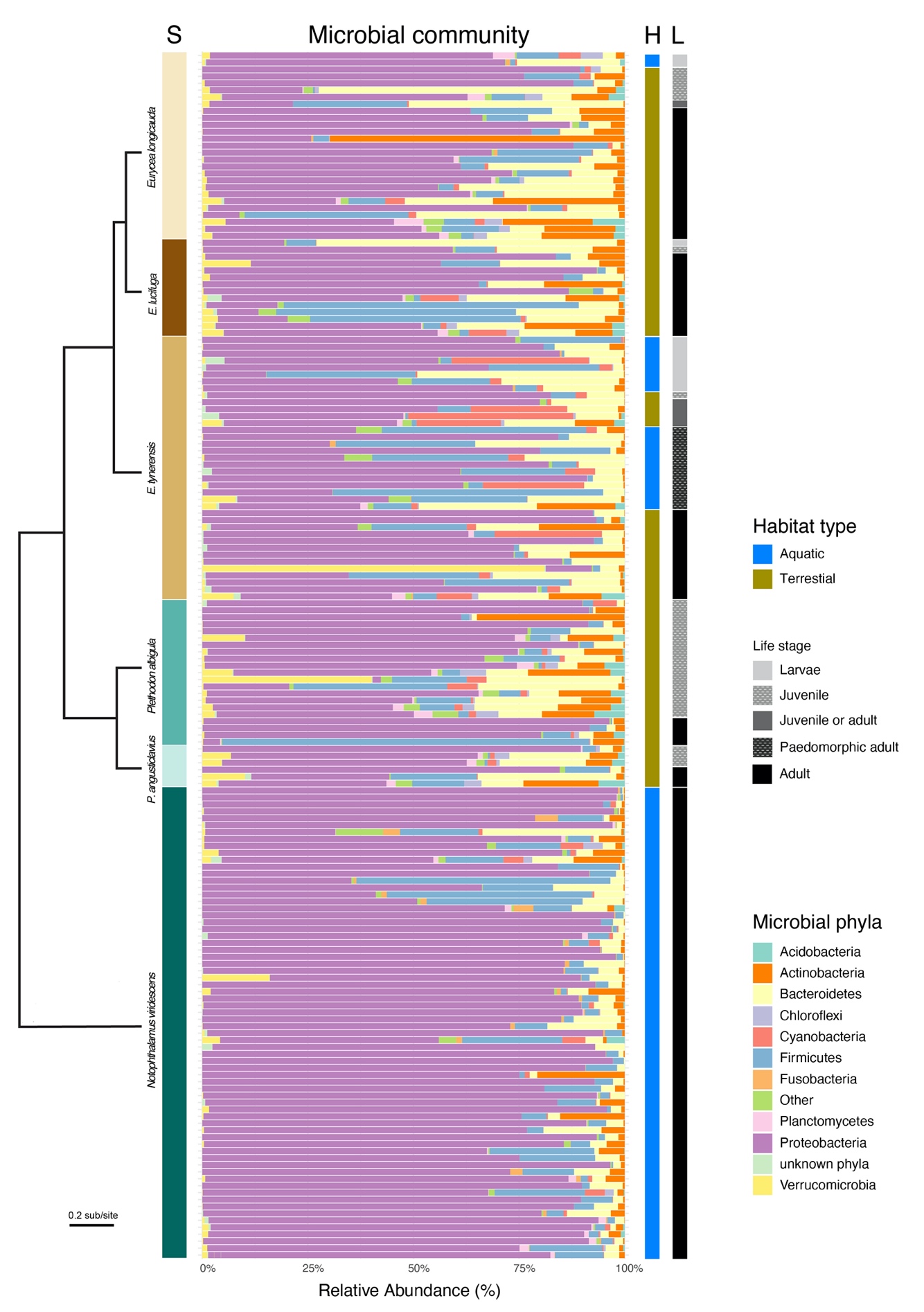


**Supplementary Figure 4.** Alpha diversity metrics Observed ASVs, Shannon-Wiener index values, and Inverse Simpson values across all six salamander species.

**
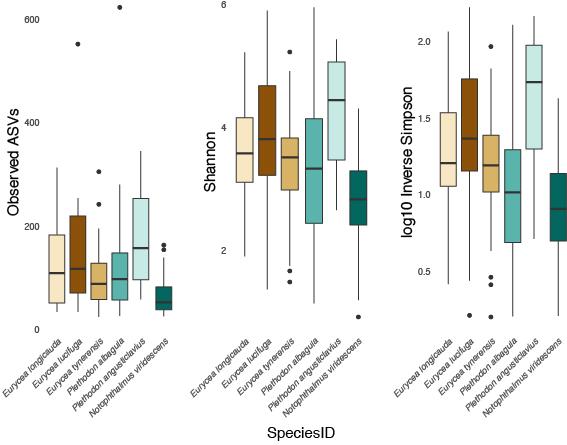
**

**Supplementary Figure 5.** CART analysis of the drivers of difference in the Shannon-Wiener Diversity Index. Host family is the most important determinant of microbial diversity, with the average precipitation of the month as a secondary driver of skin microbial diversity within the family Salamandridae.

**
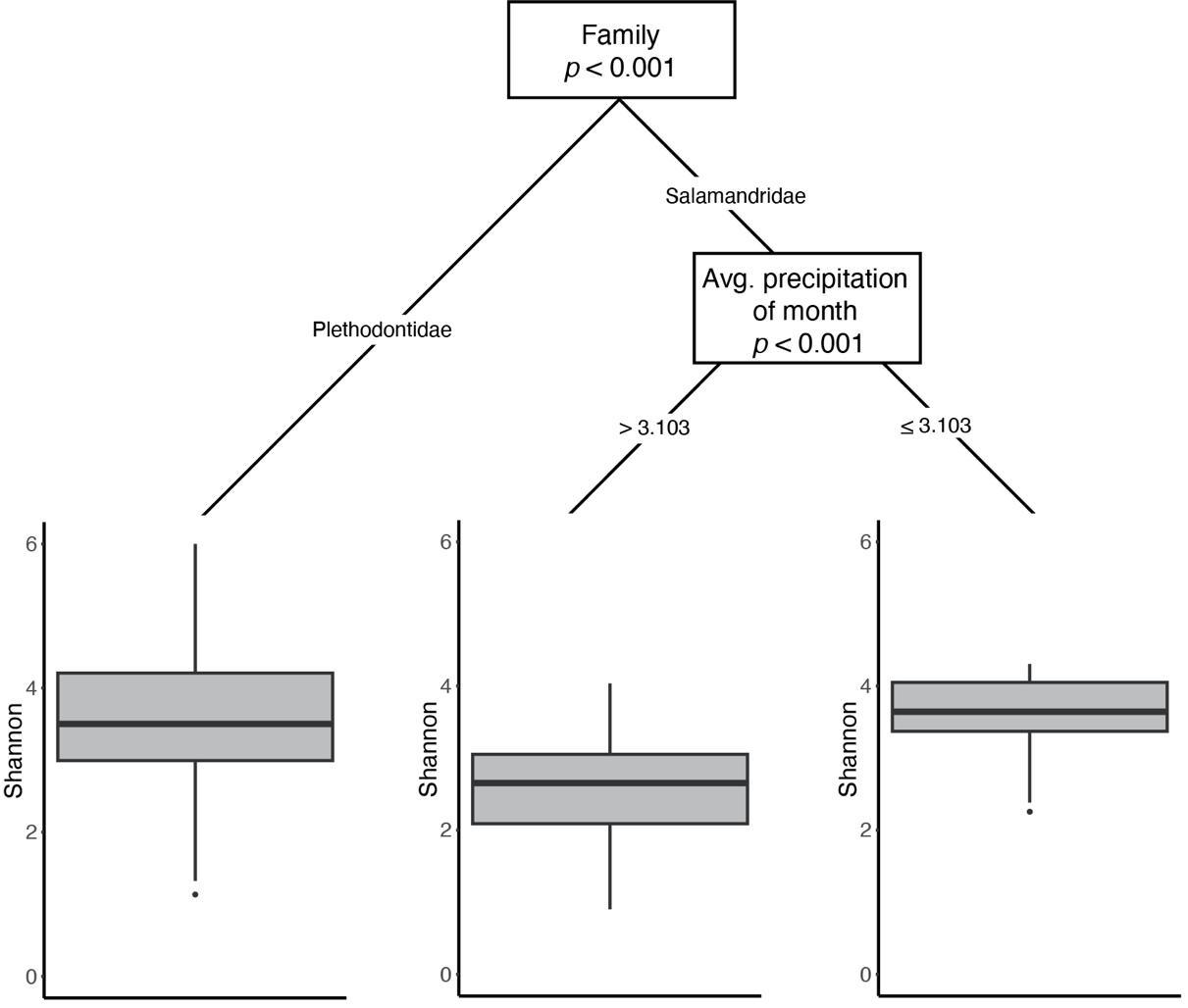
**

**Supplementary Figure 6.** CART analysis of the drivers of differences in the Inverse Simpson Diversity Index. In contrast to the other two CART analyses of alpha diversity (Fig. 4, Supplementary Fig. 2), two species (*E. lucifuga* and *P. angusticlavius*) were the most different, with family as a secondary factor. Similar to the two other indices, average precipitation of the month is an important driver of skin microbial diversity within family Salamandridae.

**
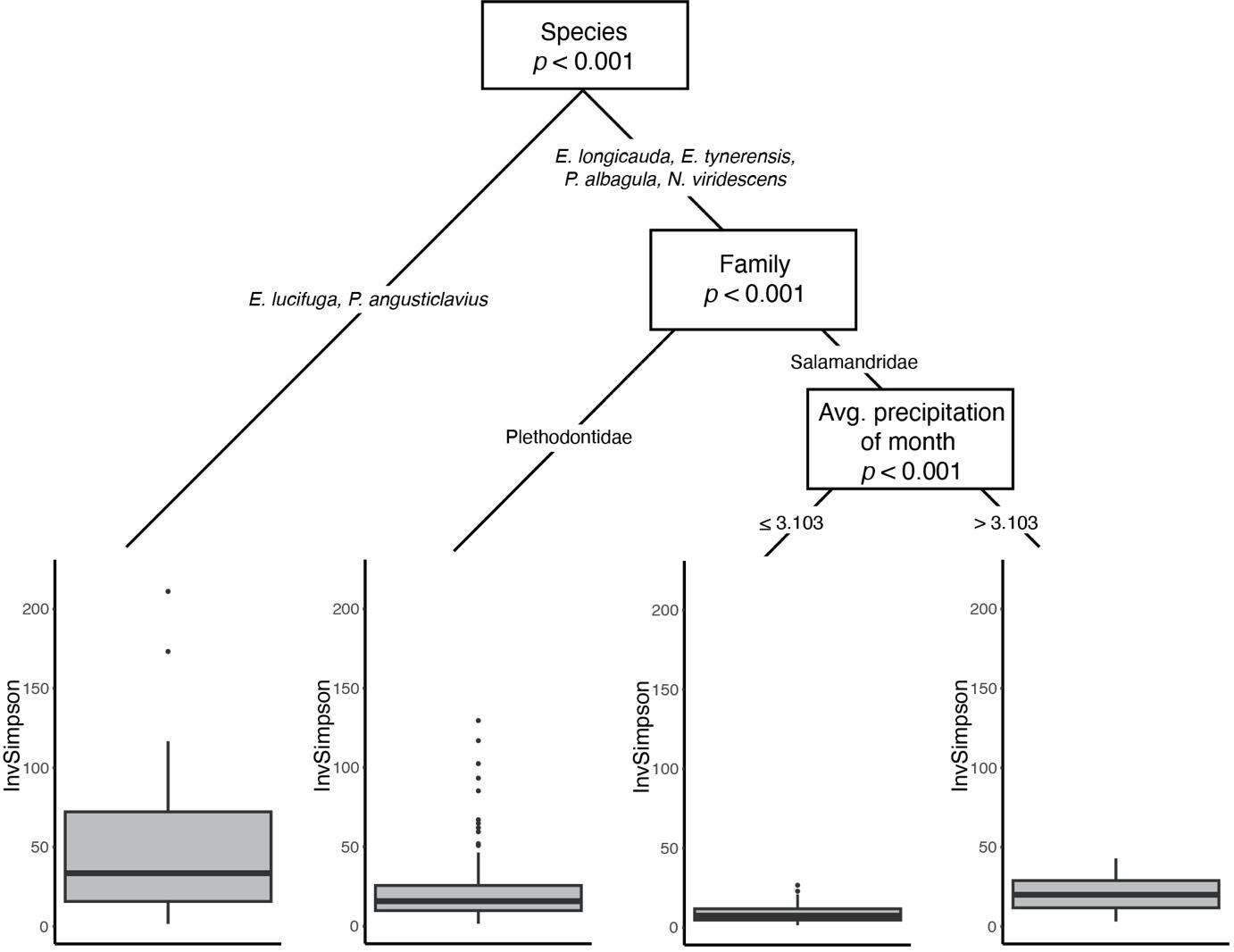
**

## Supplementary Tables

**Supplementary Table 1.** Sample distribution before and after (in parentheses) rarefaction to 1000 sequences shown across species for the four level III ecoregions in Oklahoma. While newts (Family Salamandridae) were found across all four ecoregions, the other species (all family Plethodontidae) were each found in 1–2. Sample counts after rarefaction shown

| **Species** | **Arkansas Valley** | **Boston Mountains** | **Ouachita Mountains** | **Ozark Highlands** |
| --- | --- | --- | --- | --- |
| *Notophthalmus viridescens* | 13 (11) | 10 (7) | 18 (17) | 55 (36) |
| *Eurycea longicauda* | **–** | **–** | **–** | 40 (28) |
| *E. lucifuga* | **–** | **–** | **–** | 24 (14) |
| *E. tynerensis* | **–** | 12 (7) | **–** | 52 (32) |
| *Plethodon albagula* | **–** | 2 (1) | **–** | 37 (20) |
| *P. angusticlavius* | **–** | 9 (3) | **–** | 8 (3) |

**Supplementary Table 2.** Differentially abundant microbes between salamander families Plethodontidae and Salamandridae based upon DESeq2 analyses. Microbes identified to species or strain when available. Microbes with a negative log_2_ fold change are more abundant in Plethodontidae, and those with a positive log_2_ fold change are more abundant in Salamandridae.

| **Family** | **Genus** | **Species** | **Base Mean** | **log_2_ Fold Change** | **Standard Error** | **Test Statistic** | ***p*-value** | **Adjusted *p*-value** |
| --- | --- | --- | --- | --- | --- | --- | --- | --- |
| Moraxellaceae | *Acinetobacter* | NEGJ | 18.527 | 18.527 | -4.254 | 0.582 | 3.93E-09 | 1.51E-07 |
| Ralstonia | *Polynucleobacter* | *sphagniphilus* | 6.08277858 | 6.083 | 3.965 | 0.720 | 5.32E-09 | 1.82E-07 |
| Bacteroidaceae | *Bacteroides* | unknown | 2.27424302 | 2.274 | -4.672 | 1.307 | 0.001 | 0.011 |
| Desulfovibrionaceae | *Desulfovibrio* | unknown | 1.58544499 | 1.585 | -4.107 | 1.378 | 0.004 | 0.037 |
| Bacteroidaceae | *Bacteroides* | unknown | 1.89690959 | 1.897 | -4.427 | 1.229 | 0.0007 | 0.008 |
| Moraxellaceae | *Acinetobacter* | LSZI | 17.0917601 | 17.092 | 3.431 | 0.660 | 1.63E-07 | 5.07E-06 |
| Pseudomonadaceae | *Pseudomonas* | *graminis* | 6.57241577 | 6.572 | -4.529 | 0.736 | 2.85E-07 | 8.08E-06 |
| Pseudomonadaceae | *Pseudomonas* | *peli* | 3.7832676 | 3.783 | -5.056 | 1.064 | 3.77E-05 | 0.0007 |
| Pseudomonadaceae | *Pseudomonas* | *thivervalensis* | 4.69798566 | 4.698 | -3.941 | 0.757 | 7.01E-06 | 0.0001 |
| Micrococcaceae | *Pseudarthrobacter* | *oxydans* | 3.39662443 | 3.397 | -2.845 | 0.820 | 0.002 | 0.018 |
| Alcaligenaceae | *Bordetella* | unknown | 3.03726309 | 3.037 | -5.012 | 1.251 | 0.0004 | 0.006 |
| Flavobacteriaceae | *Flavobacterium* | PEFE | 3.76047914 | 3.760 | -2.889 | 0.804 | 0.002 | 0.018 |
| Pseudomonadaceae | *Pseudomonas* | *batumici* | 14.8093713 | 14.809 | -2.364 | 0.534 | 8.06E-05 | 0.001 |
| Porphyromonadaceae | *Parabacteroides* | unknown | 1.87843545 | 1.878 | -4.418 | 1.088 | 0.0001 | 0.002 |
| Comamonadaceae | *JN679217* | DQ520187 | 3.66465562 | 3.665 | 5.456 | 0.875 | 9.22E-12 | 5.46E-10 |
| Oxalobacteraceae | *Undibacterium* | HQ111154 | 39.2082065 | 39.208 | 4.891 | 0.578 | 1.05E-16 | 1.14E-14 |
| Comamonadaceae | AF418942 | AF418942 | 3.41695755 | 3.417 | 1.821 | 0.708 | 0.005 | 0.044 |
| Comamonadaceae | AF418942 | AF418942 | 2.06184352 | 2.062 | -3.670 | 1.083 | 0.003 | 0.027 |
| Pseudomonadaceae | *Pseudomonas* | *coleopterorum* | 3.39058618 | 3.391 | -3.252 | 0.766 | 0.0001 | 0.002 |
| Fusobacteriaceae | *Cetobacterium* | *somerae* | 2.37278294 | 2.373 | 4.162 | 1.194 | 6.30E-05 | 0.001 |
| Comamonadaceae | *Rhizobacter* | unknown | 87.9024838 | 87.902 | 8.830 | 0.404 | 1.88E-75 | 6.13E-73 |
| Peptostreptococcaceae | *Romboutsia* | *sedimentorum* | 17.6017191 | 17.602 | 4.105 | 0.624 | 2.81E-11 | 1.41E-09 |
| Oxalobacteraceae | *Duganella* | FODC | 2.86788606 | 2.868 | -4.810 | 0.794 | 7.28E-08 | 2.37E-06 |
| Alishewanella | *Rheinheimera* | LXWK | 1.77183201 | 1.772 | -4.324 | 1.060 | 0.0001 | 0.002 |
| Flavobacteriaceae | *Flavobacterium* | *fluvii* | 1.34230097 | 1.342 | -3.925 | 1.088 | 0.0005 | 0.006 |
| Oxalobacteraceae | *Herbaspirillum* | *rhizosphaerae* | 2.3329191 | 2.333 | 2.969 | 0.892 | 0.00013816 | 0.00225206 |
| Neisseriaceae | *Vogesella* | *mureinivorans* | 14.8678164 | 14.868 | 5.944 | 0.664 | 9.45E-18 | 1.23E-15 |
| Neisseriaceae | *Deefgea* | *chitinilytica* | 6.12877553 | 6.129 | -3.979 | 0.956 | 0.0005 | 0.007 |
| Comamonadaceae | *Sphaerotilus* | *hippei* | 0.99696528 | 0.997 | 2.862 | 1.304 | 0.005 | 0.044 |
| Oxalobacteraceae | *Massilia* | *eurypsychrophila* | 2.95001834 | 2.950 | -4.281 | 0.868 | 1.01E-05 | 0.0002 |
| Oxalobacteraceae | *Undibacterium* | *aquatile* | 12.4692314 | 12.469 | 4.422 | 0.615 | 4.09E-13 | 3.81E-11 |
| Flavobacteriaceae | *Flavobacterium* | EU431729 | 1.11151719 | 1.112 | -3.683 | 1.087 | 0.0007 | 0.009 |
| Comamonadaceae | *Acidovorax* | DF238959 | 1.30893655 | 1.309 | -3.399 | 0.920 | 0.0003 | 0.004 |
| Comamonadaceae | *Albidiferax* | NOXW | 1.98207903 | 1.982 | 1.983 | 0.817 | 0.005 | 0.042 |
| Comamonadaceae | *Acidovorax* | *defluvii* | 2.89791811 | 2.898 | 3.145 | 0.763 | 4.36E-06 | 0.0001 |
| Pseudomonadaceae | *Pseudomonas* | *jessenii* | 22.9283762 | 22.928 | -2.130 | 0.563 | 0.0007 | 0.008 |
| Clostridiaceae | *Clostridium* | unknown | 3.47435959 | 3.474 | 4.814 | 0.907 | 4.48E-09 | 1.62E-07 |
| Pseudomonadaceae | *Pseudomonas* | LMOA | 1.81293501 | 1.813 | -2.911 | 0.992 | 0.005 | 0.044 |
| Comamonadaceae | *Rhizobacter* | unknown | 40.6450917 | 40.645 | 8.413 | 0.579 | 3.36E-35 | 7.31E-33 |
| Comamonadaceae | *Rhodoferax* | CP019236 | 2.47601431 | 2.476 | 2.666 | 0.780 | 9.80E-05 | 0.002 |
| Comamonadaceae | *Acidovorax* | *valerianellae* | 1.32046423 | 1.320 | -3.264 | 1.016 | 0.002 | 0.018 |
| Peptostreptococcaceae | *Paraclostridium* | *benzoelyticum* | 2.01696681 | 2.017 | 3.900 | 0.722 | 1.03E-09 | 4.22E-08 |
| Fusobacteriaceae | GQ850557 | GQ850557 | 1.80243482 | 1.802 | 4.217 | 1.029 | 1.12E-06 | 3.03E-05 |
| Flavobacteriaceae | *Flavobacterium* | *buctense* | 2.6001516 | 2.600 | 4.334 | 1.083 | 5.18E-06 | 0.0001 |
| Moraxellaceae | *Acinetobacter* | NEFZ | 9.03817308 | 9.038 | 1.733 | 0.579 | 0.002 | 0.017 |
| Moraxellaceae | *Acinetobacter* | NGCT | 12.3437496 | 12.344 | 4.149 | 0.897 | 6.74E-06 | 0.0001 |
| Bacillaceae | *Bacillus* | *muralis* | 1.13294281 | 1.133 | -2.875 | 0.950 | 0.002 | 0.023 |
| Moraxellaceae | *Acinetobacter* | *celticus* | 2.17792052 | 2.178 | 5.347 | 1.490 | 0.004 | 0.038 |
| Moraxellaceae | *Acinetobacter* | NHRO | 3.21961319 | 3.220 | 5.969 | 1.397 | 0.00113855 | 0.012 |
| Pseudomonadaceae | *Pseudomonas* | *granadensis* | 25.3014969 | 25.301 | -2.473 | 0.481 | 5.59E-06 | 0.0001 |
| Comamonadaceae | *Variovorax* | OCMW | 1.17627763 | 1.176 | -3.274 | 1.068 | 0.002 | 0.021 |
| Lachnospiraceae | *Epulopiscium* | unknown | 1.164105 | 1.164 | 3.802 | 1.367 | 0.0004 | 0.006 |
| Comamonadaceae | *Comamonas* | *thiooxydans* | 1.55640094 | 1.556 | 3.438 | 1.462 | 0.005 | 0.041 |
| Erwiniaceae | *Erwinia* | *endophytica* | 2.53640765 | 2.536 | -2.986 | 0.914 | 0.003 | 0.031 |
| Pseudomonadaceae | *Pseudomonas* | PDJN | 18.2294931 | 18.229 | -2.379 | 0.618 | 0.0006 | 0.008 |
| Methylophilaceae | HQ827934 | LN794158 | 0.8088923 | 0.809 | 3.315 | 1.213 | 0.0003 | 0.004 |
| Pseudomonadaceae | *Pseudomonas* | *lurida* | 23.535501 | 23.536 | -2.171 | 0.564 | 0.0006 | 0.007 |
| Methylophilaceae | JCKJ | FJ665204 | 6.28565559 | 6.286 | 6.806 | 1.304 | 1.35E-05 | 0.0003 |
| Comamonadaceae | *Sphaerotilus* | *hippei* | 1.1982487 | 1.198 | 3.658 | 1.640 | 0.006 | 0.050 |
| Comamonadaceae | *Comamonas* | BAEC | 1.52614334 | 1.526 | 3.594 | 1.398 | 0.002 | 0.019 |
| Neisseriaceae | *Aquitalea* | *pelogenes* | 6.0863584 | 6.086 | 5.841 | 1.015 | 1.16E-06 | 3.03E-05 |
| Neisseriaceae | *Vogesella* | *fluminis* | 14.7336597 | 14.734 | 4.840 | 0.727 | 2.70E-11 | 1.41E-09 |
| Flavobacteriaceae | *Flavobacterium* | *succinicans* | 7.7378491 | 7.738 | -5.467 | 0.722 | 7.49E-10 | 3.25E-08 |
| Neisseriaceae | *Aquitalea* | *denitrificans* | 7.63580201 | 7.636 | 9.207 | 0.859 | 2.58E-12 | 2.10E-10 |
| Pseudomonadaceae | *Pseudomonas* | *endophytica* | 13.5556679 | 13.556 | -3.213 | 0.569 | 1.48E-06 | 3.72E-05 |
| Comamonadaceae | *Limnohabitans* | CP011834 | 1.12065639 | 1.121 | 3.804 | 1.308 | 0.0002 | 0.003 |
| Microbacteriaceae | *Rathayibacter* | unknown | 3.75162597 | 3.752 | -5.170 | 1.254 | 0.0005 | 0.007 |
| Flavobacteriaceae | *Flavobacterium* | *terrigena* | 2.80948788 | 2.809 | -4.874 | 0.825 | 2.47E-07 | 7.31E-06 |
| Comamonadaceae | *Ideonella* | *paludis* | 3.3694293 | 3.369 | 4.924 | 0.846 | 1.80E-10 | 8.40E-09 |
| Bacteroidaceae | *Bacteroides* | unknown | 1.57275744 | 1.573 | -4.171 | 1.301 | 0.002 | 0.019 |
| Alcaligenaceae | *Orrella* | unknown | 0.59123314 | 0.591 | 2.894 | 1.315 | 0.002 | 0.023 |
| Pseudomonadaceae | *Pseudomonas* | *umsongensis* | 8.66532344 | 8.665 | -2.695 | 0.690 | 0.0006 | 0.008 |
| Peptostreptococcaceae | *Romboutsia* | EU089965 | 12.0860824 | 12.086 | 6.436 | 0.587 | 1.34E-26 | 2.18E-24 |
| Porphyromonadaceae | *Parabacteroides* | unknown | 2.15633226 | 2.156 | -4.606 | 1.153 | 0.0002 | 0.003 |
| Comamonadaceae | *Curvibacter* | *gracilis* | 6.14789315 | 6.148 | 5.293 | 0.787 | 3.98E-12 | 2.89E-10 |
| Comamonadaceae | *Leptothrix* | *discophora* | 8.32534066 | 8.325 | 5.200 | 0.784 | 8.11E-12 | 5.29E-10 |
| Flavobacteriaceae | *Flavobacterium* | *fluminis* | 1.46359032 | 1.464 | -3.352 | 0.987 | 0.0009 | 0.011 |
| Comamonadaceae | *Rhizobacter* | unknown | 361.06383 | 361.064 | 9.432 | 0.400 | 2.35E-84 | 1.53E-81 |
| Bacteroidaceae | *Bacteroides* | unknown | 1.89784262 | 1.898 | -4.257 | 1.205 | 0.0008 | 0.010 |

**Supplementary Table 3:** Differentially abundant microbes between salamander families Plethodontidae and Salamandridae based upon ANCOM-BC. Microbes identified to species or strain when available. Higher LogFoldChanges represent microbial taxa with greater abundance in family Salamandridae, and lower ones have greater abundance in family Plethodontidae.

| ***Taxon*** | **log_2_ Fold Change** | **Standard Error** | ***p*-value** | **Adjusted *p*-value** | **Test Statistic** |
| --- | --- | --- | --- | --- | --- |
| *Acidovorax.defluvii* | 1.5572415 | 0.17372207 | 3.13E-19 | 6.17E-17 | 8.96398208 |
| *Aquitalea.denitrificans* | 1.46271277 | 0.17321795 | 3.06E-17 | 5.96E-15 | 8.44434852 |
| *Aquitalea.pelogenes* | 1.28722227 | 0.19440947 | 3.56E-11 | 6.70E-09 | 6.62119132 |
| *Arcobacter.defluvii* | 0.57105558 | 0.14847907 | 0.00012005 | 0.01620608 | 3.84603413 |
| *Bacillus.muralis* | -0.4172384 | 0.10750673 | 0.00010401 | 0.01414521 | -3.8810443 |
| *BAEC_s* | 0.73578474 | 0.14513822 | 3.99E-07 | 6.94E-05 | 5.06954507 |
| *Cetobacterium.somerae* | 0.85901924 | 0.14415181 | 2.54E-09 | 4.59E-07 | 5.95912923 |
| *Comamonas.jiangduensis* | 0.49677654 | 0.12728179 | 9.50E-05 | 0.01301787 | 3.90296625 |
| *Comamonas.thiooxydans* | 0.69885522 | 0.15565451 | 7.13E-06 | 0.00115498 | 4.48978467 |
| *CP019236_s* | 0.82948324 | 0.17008171 | 1.08E-06 | 0.00018422 | 4.87696919 |
| *Curvibacter.gracilis* | 1.53306409 | 0.17111737 | 3.27E-19 | 6.41E-17 | 8.95913764 |
| *Delftia.acidovorans* | 0.47645669 | 0.11862238 | 5.90E-05 | 0.00826671 | 4.01658361 |
| *DF238959_s* | -0.3449981 | 0.08982855 | 0.00012272 | 0.01644441 | -3.840629 |
| *DQ520187_s* | 1.34282613 | 0.16432493 | 3.04E-16 | 5.90E-14 | 8.17177376 |
| *EU089965_s* | 2.33914268 | 0.19452291 | 2.63E-33 | 5.33E-31 | 12.025024 |
| *EU431729_s* | -0.403847 | 0.09430198 | 1.85E-05 | 0.00286464 | -4.2824876 |
| *Exiguobacterium.acetylicum* | 0.62780103 | 0.15531437 | 5.30E-05 | 0.0075214 | 4.04213095 |
| *FJ665204_s* | 0.91971108 | 0.21005423 | 1.20E-05 | 0.0019005 | 4.37844597 |
| *Flavobacterium.buctense* | 0.75720511 | 0.18907806 | 6.21E-05 | 0.00863056 | 4.00472226 |
| *Flavobacterium.chungnamense* | 0.55028626 | 0.12348461 | 8.34E-06 | 0.00134243 | 4.45631466 |
| *Flavobacterium.fluvii* | -0.4237617 | 0.0999005 | 2.22E-05 | 0.00339196 | -4.2418381 |
| *Flavobacterium.succinicans* | -0.886633 | 0.1393294 | 1.97E-10 | 3.67E-08 | -6.3635748 |
| *Flavobacterium.terrigena* | -0.7188128 | 0.1191253 | 1.60E-09 | 2.91E-07 | -6.0340901 |
| *FODC_s* | -0.5728725 | 0.10613471 | 6.75E-08 | 1.20E-05 | -5.3975986 |
| *GQ850557_s* | 0.98071463 | 0.14606497 | 1.89E-11 | 3.57E-09 | 6.71423551 |
| *Hafnia.alvei* | 0.84385073 | 0.20728795 | 4.68E-05 | 0.0069308 | 4.07091068 |
| *Herbaspirillum.aquaticum* | 0.36053004 | 0.09986212 | 0.00030587 | 0.03915122 | 3.6102781 |
| *Herbaspirillum.rhizosphaerae* | 0.80993807 | 0.15275207 | 1.14E-07 | 2.01E-05 | 5.30230487 |
| *HQ111154_s* | 2.33723106 | 0.23561323 | 3.42E-23 | 6.86E-21 | 9.91977868 |
| *Ideonella.paludis* | 1.30730323 | 0.17448362 | 6.76E-14 | 1.30E-11 | 7.49241239 |
| *JX224059_s* | 0.857804 | 0.19032069 | 6.57E-06 | 0.00107098 | 4.50715062 |
| *JZUE_s* | 0.56129998 | 0.13464349 | 3.06E-05 | 0.004624 | 4.16878676 |
| *Leptothrix.discophora* | 1.97256188 | 0.20385647 | 3.80E-22 | 7.53E-20 | 9.67622899 |
| *LMLM_s* | 0.74735741 | 0.20327921 | 0.00023645 | 0.03097488 | 3.67650691 |
| *LN794158_s* | 0.85698456 | 0.13754416 | 4.65E-10 | 8.60E-08 | 6.23061412 |
| *LXWK_s* | -0.5121697 | 0.10818205 | 2.20E-06 | 0.00036923 | -4.7343314 |
| *Massilia.eurypsychrophila* | -0.5533558 | 0.12168747 | 5.43E-06 | 0.00090179 | -4.5473527 |
| *Massilia.glaciei* | -0.7034112 | 0.17290257 | 4.74E-05 | 0.00696297 | -4.0682519 |
| *Moraxella.osloensis* | 0.5445809 | 0.11417393 | 1.84E-06 | 0.00031173 | 4.76974832 |
| *MQMM_s* | 0.7076665 | 0.17319917 | 4.39E-05 | 0.00654334 | 4.08585381 |
| *NEFZ_s* | 0.69600508 | 0.19632644 | 0.0003924 | 0.04944265 | 3.54514177 |
| *NEGJ_s* | -0.8799222 | 0.1747485 | 4.77E-07 | 8.25E-05 | -5.0353638 |
| *NGCT_s* | 0.74857701 | 0.19619238 | 0.00013589 | 0.01793794 | 3.81552533 |
| *NHRO_s* | 0.74482814 | 0.16401772 | 5.59E-06 | 0.00092317 | 4.54114424 |
| *NOXW_s* | 0.81851865 | 0.13993906 | 4.94E-09 | 8.90E-07 | 5.84910776 |
| *Paraclostridium.benzoelyticum* | 1.03246731 | 0.16939522 | 1.09E-09 | 2.00E-07 | 6.09502022 |
| *PEFE_s* | -0.4457032 | 0.12350559 | 0.00030765 | 0.03915122 | -3.6087692 |
| *Pelomonas.aquatica* | 0.56447829 | 0.13236378 | 2.00E-05 | 0.00308404 | 4.26459804 |
| *Polynucleobacter.sphagniphilus* | 1.79106708 | 0.18508809 | 3.78E-22 | 7.53E-20 | 9.67683594 |
| *Pseudomonas.coleopterorum* | -0.4925471 | 0.11816364 | 3.07E-05 | 0.004624 | -4.1683478 |
| *Pseudomonas.graminis* | -0.6934213 | 0.11172372 | 5.42E-10 | 9.96E-08 | -6.2065718 |
| *Pseudomonas.granadensis* | -1.1489285 | 0.17968445 | 1.61E-10 | 3.02E-08 | -6.3941458 |
| *Pseudomonas.lurida* | -0.6823861 | 0.18736037 | 0.00027042 | 0.03488388 | -3.642105 |
| *Pseudomonas.peli* | -0.6486551 | 0.11970856 | 6.01E-08 | 1.07E-05 | -5.4186188 |
| *Pseudomonas.thivervalensis* | -0.6387837 | 0.11175841 | 1.09E-08 | 1.96E-06 | -5.7157546 |
| *Romboutsia.sedimentorum* | 2.37510348 | 0.23567338 | 6.92E-24 | 1.40E-21 | 10.0779457 |
| *Shigella.flexneri* | 0.65541251 | 0.15040278 | 1.31E-05 | 0.00207655 | 4.3577153 |
| *Sphaerotilus.hippei* | 0.95102703 | 0.180955 | 1.48E-07 | 2.58E-05 | 5.25559973 |
| *Tolumonas.auensis* | 0.55221151 | 0.11355061 | 1.16E-06 | 0.00019642 | 4.86313095 |
| *Undibacterium.aquatile* | 1.51659294 | 0.21901576 | 4.37E-12 | 8.35E-10 | 6.92458345 |
| *unk_S.13* | -0.5976829 | 0.13946721 | 1.82E-05 | 0.00284468 | -4.2854725 |
| *unk_S.16* | -0.4700371 | 0.11694874 | 5.84E-05 | 0.00823483 | -4.0191719 |
| *unk_S.18* | -0.8906583 | 0.18944032 | 2.58E-06 | 0.00043124 | -4.7015244 |
| *unk_S.19* | 0.48434795 | 0.11953589 | 5.08E-05 | 0.00736637 | 4.05190392 |
| *unk_S.26* | -0.472012 | 0.12852165 | 0.00024007 | 0.03120912 | -3.6726266 |
| *unk_S.28* | -0.5931161 | 0.13624541 | 1.34E-05 | 0.0021055 | -4.3532923 |
| *unk_S.29* | 1.11007131 | 0.16263496 | 8.76E-12 | 1.66E-09 | 6.82553946 |
| *unk_S.31* | -0.5228813 | 0.13667047 | 0.00013032 | 0.01733247 | -3.8258544 |
| *unk_S.33* | -0.5373604 | 0.12199543 | 1.06E-05 | 0.00169443 | -4.4047584 |
| *unk_S.34* | -0.5105921 | 0.1259163 | 5.01E-05 | 0.00731923 | -4.0550121 |
| *unk_S.36* | -0.4847778 | 0.12290629 | 8.00E-05 | 0.01104517 | -3.944288 |
| *unk_S.38* | 0.90501753 | 0.20035584 | 6.27E-06 | 0.00102839 | 4.5170509 |
| *unk_S.44* | -0.4447281 | 0.1099402 | 5.23E-05 | 0.0074764 | -4.0451819 |
| *unk_S.45* | -0.4809454 | 0.11882025 | 5.17E-05 | 0.00744906 | -4.047672 |
| *unk_S.53* | -0.5668542 | 0.13466102 | 2.56E-05 | 0.00389041 | -4.20949 |
| *unk_S.8* | 0.56128598 | 0.11250741 | 6.07E-07 | 0.00010446 | 4.9888803 |
| *unk_S.9* | 5.32928902 | 0.23339609 | 2.12E-115 | 4.33E-113 | 22.8336691 |
| *Vogesella.fluminis* | 1.84185935 | 0.23436838 | 3.88E-15 | 7.48E-13 | 7.85882174 |
| *Vogesella.mureinivorans* | 2.2399881 | 0.22779676 | 8.09E-23 | 1.62E-20 | 9.8332746 |

**Supplementary Table 4.** Differences in alpha diversity of salamander microbiomes by host phylogenetic group (i.e., families, genera, and species). Differences by family were assessed using the Kruskal-Wallis test, whereas those by genera and species were analyzed using a pairwise-Wilcoxon rank sum test with Holm *p*-value correction (bold values signify *p*-values <0.05; chi-squared values or effect sizes shown between each *p*-value). The results indicate that microbial alpha diversity differs by salamander family, a pattern evident in all subsequent comparisons between *Notophthalmus* (family Salamandridae) and the other genera and species (all family Plethodontidae).

| **Comparison** | |  | **ASVs** | **Shannon** | **Inv. Simpson** |
| --- | --- | --- | --- | --- | --- |
| **Family** | Plethodontidae vs. Salamandridae | | **1.66E-07** | **1.09E-07** | **0.002** |
|  | *chi-*squared | | 27.396 | 28.200 | 9.574 |
| **Genus** | *Eurycea* vs. *Notophthalmus* | | **5.60E-06** | **2.90E-07** | **1.50E-06** |
|  | effect size | | 0.387 | 0.433 | 0.408 |
|  | *Plethodon* vs. *Eurycea* | | 0.514 | 0.809 | 0.580 |
|  | effect size | | 0.0632 | 0.024 | 0.054 |
|  | *Plethodon* vs. *Notophthalmus* | | **0.0002** | **0.009** | **0.040** |
|  | effect size | | 0.388 | 0.286 | 0.236 |
| **Species** | *E. longicauda* vs. *E. tynerensis* | | 1 | 1 | 1 |
|  | effect size | | 0.137 | 0.115 | 0.089 |
|  | *E. longicauda* vs. *N. viridescens* | | **0.003** | **0.0001** | **0.0005** |
|  | effect size | | 0.374 | 0.442 | 0.416 |
|  | *E. longicauda* vs. *P. albagula* | | 1 | 1 | 1 |
|  | effect size | | 0.042 | 0.173 | 0.216 |
|  | *E. longicauda* vs. *P. angusticlavius* | | 1 | 1 | 1 |
|  | effect size | | 0.205 | 0.201 | 0.271 |
|  | *E. lucifuga* vs. *E. longicauda* | | 1 | 1 | 1 |
|  | effect size | | 0.076 | 0.021 | 0.128 |
|  | *E. lucifuga* vs. *N. viridescens* | | **0.010** | **0.044** | **0.044** |
|  | effect size | | 0.369 | 0.319 | 0.319 |
|  | *E. lucifuga* vs. *P. albagula* | | 1 | 1 | 1 |
|  | effect size | | 0.145 | 0.171 | 0.239 |
|  | *E. lucifuga* vs. *P. angusticlavius* | | 1 | 1 | 1 |
|  | effect size | | 0.166 | 0.166 | 0.203 |
|  | *E. tynerensis* vs. *E. lucifuga* | | 1 | 1 | 1 |
|  | effect size | | 0.176 | 0.163 | 0.197 |
|  | *E. tynerensis* vs. *N. viridescens* | | **0.013** | **0.001** | **0.004** |
|  | effect size | | 0.313 | 0.376 | 0.345 |
|  | *E. tynerensis* vs. *P. albagula* | | 1 | 1 | 1 |
|  | effect size | | 0.088 | 0.059 | 0.127 |
|  | *E. tynerensis* vs. *P. angusticlavius* | | 0.796 | 0.993 | 0.691 |
|  | effect size | | 0.264 | 0.249 | 0.279 |
|  | *N. viridescens* vs. *P. albagula* | | **0.028** | 0.649 | 1 |
|  | effect size | | 0.318 | 0.197 | 0.136 |
|  | *N. viridescens* vs. *P. angusticlavius* | | **0.035** | **0.045** | **0.045** |
|  | effect size | | 0.337 | 0.331 | 0.331 |
|  | *P. albagula* vs. *P. angusticlavius* | | 1 | 0.993 | 0.691 |
|  | effect size | | 0.225 | 0.314 | 0.359 |

**Supplementary Table 5.** Role of pathogen status and ecoregion on alpha diversity within family Salamandridae (i.e., newts). The impact of ranavirus (RV) and *Batrachochytrium dendrobatidis* (*Bd*) status (i.e., presence/absence) was evaluated with Kruskal-Wallis tests; significance of ecoregion was assessed using a pairwise-Wilcoxon rank sum test with Holm *p*-value correction (no p-values <0.05; chi-squared or effect sizes shown between each p-value). When considering factors separately, we found no significant differences between disease status, or between ecoregions in any alpha diversity metric.

| *Family Salamandridae* | |  | **ASVs** | **Shannon** | **Inverse Simpson** |
| --- | --- | --- | --- | --- | --- |
| **RV** | yes vs. no | | 0.808 | 0.696 | 0.958 |
|  | *chi-*squared | | 0.059 | 0.153 | 0.003 |
| ***Bd*** | yes vs. no | | 0.306 | 0.455 | 0.619 |
|  | *chi-*squared | | 1.048 | 0.558 | 0.248 |
| **Ecoregion** | Arkansas Valley vs. Boston Mountains | | 0.417 | 1 | 1 |
|  | effect size | | 0.395 | 0.139 | 0.139 |
|  | Arkansas Valley vs. Ozark Highlands | | 0.435 | 1 | 1 |
|  | effect size | | 0.214 | 0.022 | 0.077 |
|  | Arkansas Valley vs. Ouachita Mountains | | 0.435 | 0.63 | 1 |
|  | effect size | | 0.258 | 0.271 | 0.218 |
|  | Boston Mountains vs. Ozark Highlands | | 0.554 | 0.54 | 0.67 |
|  | effect size | | 0.093 | 0.261 | 0.246 |
|  | Boston Mtns vs. Ouachita Mountains | | 0.227 | 0.54 | 0.67 |
|  | effect size | | 0.415 | 0.344 | 0.318 |
|  | Ouachita Mountains vs. Ozark Highlands | | 0.086 | 0.63 | 1 |
|  | effect size | | 0.338 | 0.196 | 0.139 |

**Supplementary Table 6.** Role of pathogen status and ecoregion on alpha diversity within family Plethodontidae. The impact of ranavirus and *Batrachochytrium dendrobatidis* (*Bd)* status (i.e., presence/absence) was evaluated with Kruskal-Wallis tests; significance of ecoregion or habitat was assessed using a pairwise-Wilcoxon rank sum test with Holm *p*-value correction (no p-values <0.05; Chi-squared values shown below each p-value). Here we found no significant differences between pathogen status, habitat type, or between ecoregions in any of the alpha diversity metrics.

| *Family Plethodontidae* | |  | **ASVs** | **Shannon** | **Inverse Simpson** |
| --- | --- | --- | --- | --- | --- |
| **Ranavirus** | yes vs. no | | 0.47 | 0.32 | 0.3 |
|  | *chi-*squared | | 0.536 | 1 | 1.11 |
| ***Bd*** | yes vs. no | | 0.94 | 0.724 | 0.696 |
|  | *chi-*squared | | 0.001 | 0.125 | 0.153 |
| **Habitat** | aquatic vs. terrestrial | | 0.335 | 0.97 | 0.79 |
|  | effect size | | 0.931 | 0.001 | 0.071 |
| **Ecoregion** | Boston Mtns. vs. Ozark Highlands | | 0.053 | 0.097 | 0.2 |
|  | effect size | | 3.75 | 2.76 | 1.65 |

**Supplementary Table 7.** Impact of phylogenetic differentiation on beta diversity across families, genera, and species. Tests of beta diversity revealed differences in skin microbiomes among salamander families and genera, but fewer differences at the level of comparisons among species. Beta diversity was evaluated using Permutational Analysis of Variance tests with Holm *p*-value correction and permutations set to 10,000 (bold values signify p-values <0.05; *r*-squared shown between each p-valuet).

| *All Species* |  | **unweighted UniFrac** | **weighted UniFrac** |
| --- | --- | --- | --- |
| **Family** | Plethodontidae vs. Salamandridae | **0.001** | **0.001** |
|  | *r-*squared | 0.050 | 0.165 |
| **Genus** | *Plethodon* vs. *Notophthalmus* | **0.001** | **0.001** |
|  | *r-*squared | 0.065 | 0.179 |
|  | *Plethodon* vs. *Eurycea* | **0.013** | 0.064 |
|  | *r-*squared | 0.016 | 0.017 |
|  | *Eurycea* vs. *Notophthalmus* | **0.001** | **0.001** |
|  | *r-*squared | 0.050 | 0.185 |
| **Species** | *E. longicauda* vs. *E. lucifuga* | 0.348 | 0.229 |
|  | *r-*squared | 0.025 | 0.031 |
|  | *E. longicauda* vs. *E. tynerensis* | **0.004** | **0.031** |
|  | *r-*squared | 0.027 | 0.032 |
|  | *E. longicauda* vs. *N. viridescens* | **0.001** | **0.001** |
|  | *r-*squared | 0.053 | 0.210 |
|  | *E. longicauda* vs. *P. albagula* | 0.461 | 0.336 |
|  | *r-*squared | 0.021 | 0.023 |
|  | *E. longicauda* vs. *P. angusticlavius* | 0.543 | 0.939 |
|  | *r-*squared | 0.028 | 0.013 |
|  | *E. lucifuga* vs. *N. viridescens* | **0.001** | **0.001** |
|  | *r-*squared | 0.052 | 0.196 |
|  | *E. lucifuga* vs. *E. tynerensis* | **0.005** | **0.045** |
|  | *r-*squared | 0.039 | 0.040 |
|  | *E. lucifuga* vs. *P. albagula* | 0.44 | 0.101 |
|  | *r-*squared | 0.029 | 0.049 |
|  | *E. lucifuga* vs. *P. angusticlavius* | 0.354 | 0.815 |
|  | *r-*squared | 0.054 | 0.030 |
|  | *E. tynerensis* vs. *N. viridescens* | **0.001** | **0.001** |
|  | *r-*squared | 0.050 | 0.182 |
|  | *E. tynerensis* vs. *P. albagula* | **0.001** | **0.006** |
|  | *r-*squared | 0.0385 | 0.048 |
|  | *E. tynerensis* vs. *P. angusticlavius* | **0.027** | 0.349 |
|  | *r-*squared | 0.034 | 0.024 |
|  | *N. viridescens* vs. *P. albagula* | **0.001** | **0.001** |
|  | *r-*squared | 0.057 | 0.160 |
|  | *N. viridescens* vs. *P. angusticlavius* | **0.001** | **0.001** |
|  | *r-*squared | 0.038 | 0.117 |
|  | *P. albagula* vs. *P. angusticlavius* | 0.837 | 0.658 |
|  | *r-*squared | 0.030 | 0.027 |

**Supplementary Table 8.** Factors influencing beta diversity within the family Salamandridae. Significance assessed with a Permutational Analysis of Variance tests with Holm *p*-value correction and permutations set to 10,000 (bold values signify p-values <0.05; *r*-squared shown between each p-value). The weighted UniFrac test considers microbial phylogeny, while the unweighted test does not. We found there was no significant difference between disease statuses. We did find a significant difference in beta diversity between all ecoregions in at least one of the UniFrac tests.

| *Family Salamandridae* | |  | **Unweighted UniFrac** | **Weighted UniFrac** |
| --- | --- | --- | --- | --- |
| **RV** | Yes vs. No | | 0.29 | 0.15 |
|  | *r-*squared | | 0.024 | 0.033 |
| ***Bd*** | Yes vs. No | | 0.57 | 0.29 |
|  | *r*-squared | | 0.022 | 0.026 |
| **Ecoregion** | Arkansas Valley vs. Boston Mountains | | 0.053 | **0.045** |
|  | *r-*squared | | 0.063 | 0.097 |
|  | Arkansas Valley vs. Ouachita Mountains | | **0.003** | **0.023** |
|  | *r-*squared | | 0.065 | 0.074 |
|  | Arkansas Valley vs. Ozark Highlands | | **0.001** | 0.163 |
|  | *r-*squared | | 0.038 | 0.022 |
|  | Boston Mountains vs. Ouachita Mountains | | **0.002** | **0.001** |
|  | *r-*squared | | 0.009 | 0.190 |
|  | Boston Mountains vs. Ozark Highlands | | 0.211 | **0.01** |
|  | *r-*squared | | 0.018 | 0.050 |
|  | Ouachita Mountains vs. Ozark Highlands | | **0.001** | **0.009** |
|  | *r-*squared | | 0.066 | 0.042 |

**Supplementary Table 9.** Factors influencing beta diversity within the family Plethodontidae. Significance assessed with a Permutational Analysis of Variance tests with Holm *p*-value correction and permutations set to 10,000 (bold values signify p-values <0.05; *r-*squared shown between each p-value). The unweighted UniFrac test treats phylogeny as a categorical variable, while the weighted test treats it as a continuous variable. We found no significant difference in beta diversity between pathogen statuses or ecoregions. We did find significant differences between the beta diversity of the life stages and habitats in the weighted-UniFrac.

| *Family Plethodontidae* |  | **Unweighted UniFrac** | **Weighted UniFrac** |
| --- | --- | --- | --- |
| **RV** | Yes vs. No | 0.07 | 0.41 |
|  | *r-*squared | 0.017 | 0.013 |
| ***Bd*** | Yes vs. No | 0.18 | 0.47 |
|  | *r-*squared | 0.0148 | 0.012 |
| **Habitat** | aquatic vs. terrestrial | **0.015** | 0.232 |
|  | *r-*squared | 0.024 | 0.017 |
| **Ecoregion** | Ozark Highlands vs. Boston Mountains | 0.117 | 0.698 |
|  | *r-*squared | 0.020 | 0.051 |
| **Life Stage** | juvenile vs. adult | **0.038** | 0.382 |
|  | *r-*squared | 0.019 | 0.013 |
|  | juvenile vs. paedomorphic adult | **0.004** | 0.082 |
|  | *r-*squared | 0.052 | 0.043 |
|  | juvenile vs. larva | **0.006** | 0.094 |
|  | *r-*squared | 0.053 | 0.045 |
|  | adult vs. paedomorphic adult | 0.085 | 0.375 |
|  | *r-*squared | 0.02 | 0.016 |
|  | adult vs. larva | **0.012** | 0.217 |
|  | *r-*squared | 0.025 | 0.02 |
|  | paedomorphic adult vs. larva | 0.468 | **0.015** |
|  | *r-*squared | 0.044 | 0.047 |

**Supplementary Table 10.** Partial Mantel tests of the correlation between environmental conditions and beta diversity (based on Bray-Curtis distances; (bold values signify p-values <0.05). These results account for geographic distance, measured as the Euclidean distance between sampling localities.

| **Comparison** | **Variables** | **Mantel statistic *r*** | **significance** |
| --- | --- | --- | --- |
| **All Species** | elevation \| distance | 0.122 | **0.001** |
|  | annual precipitation \| distance | 0.060 | **0.005** |
|  | seasonal precipitation \| distance | 0.060 | **0.006** |
|  | monthly precipitation \| distance | 0.039 | 0.133 |
|  | annual temperature \| distance | 0.195 | **0.001** |
|  | seasonal temperature \| distance | 0.179 | **0.001** |
|  | monthly temperature \| distance | 0.209 | **0.001** |
| **Salamandridae** | elevation \| distance | 0.142 | **0.025** |
|  | annual precipitation \| distance | 0.010 | 0.402 |
|  | seasonal precipitation \| distance | -0.026 | 0.745 |
|  | monthly precipitation \| distance | 0.297 | **0.001** |
|  | annual temperature \| distance | 0.104 | **0.038** |
|  | seasonal temperature \| distance | 0.115 | **0.028** |
|  | monthly temperature \| distance | 0.264 | **0.001** |
| **Plethodontidae** | elevation \| distance | 0.111 | 0.052 |
|  | annual precipitation \| distance | 0.108 | **0.012** |
|  | seasonal precipitation \| distance | 0.166 | **0.003** |
|  | monthly precipitation \| distance | 0.214 | **0.001** |
|  | annual temp \| distance | 0.094 | **0.004** |
|  | seasonal temp \| distance | 0.145 | **0.001** |
|  | monthly temp \| distance | 0.166 | **0.002** |

**Supplementary Table 11.** Mantel tests of the correlation between environmental conditions and beta diversity (based on Bray-Curtis distances; (bold values signify p-values <0.05) between microbiomes. These correlations, without accounting for geography, are similar to those from the partial Mantel results.

| **Variables** | **Mantel statistic *r*** | ***r* = 0** | **upper limit** | **lower limit** |
| --- | --- | --- | --- | --- |
| elevation | 0.123 | **0.001** | 0.146 | 0.104 |
| annual precipitation | 0.063 | **0.002** | 0.077 | 0.050 |
| seasonal precipitation | 0.062 | **0.009** | 0.077 | 0.048 |
| monthly precipitation | 0.044 | 0.275 | 0.063 | 0.028 |
| annual temperature | 0.196 | **0.001** | 0.214 | 0.178 |
| seasonal temperature | 0.180 | **0.001** | 0.197 | 0.164 |
| monthly temperature | 0.209 | **0.001** | 0.230 | 0.189 |

**Supplementary Table 12.** Differentially abundant antifungal microbes between animals based on *Bd* status based upon DESeq2 analyses. Negative log_2_ fold change values indicate a greater abundance of that microbe in animals without *Bd*, and positive ones indicate greater abundance in animals with *Bd*.

| **Name** | **Family** | **Genus** | **Species** | **Base Mean** | **log_2_ Fold Change** | **Standard Error** | **Test Statistic** | ***p*-value** | **Adjusted *p*-value** |
| --- | --- | --- | --- | --- | --- | --- | --- | --- | --- |
| Craugastorcrassidigitus-inhibitory_91 | Pseudomonadaceae | *Pseudomonas* | unknown | 2.502 | -2.471 | 0.676 | 12.747 | 0.0003 | 0.006 |
| Dendrobatesauratus-inhibitory_2 | Pseudomonadaceae | *Pseudomonas* | *fragi* | 8.740 | -2.465 | 0.539 | 17.368 | 3.08E-05 | 0.0009 |
| Smiliscasordida-inhibitory_34 | Pseudomonadaceae | *Pseudomonas* | unknown | 1.770 | -2.862 | 0.868 | 10.926 | 0.0009 | 0.010 |
| Atelopuselegans-ns_15 | Oxalobacteraceae | *Cupriavidus* | unknown | 5.307 | -3.415 | 0.697 | 19.406 | 1.06E-05 | 0.0005 |
| Colostethuspanamensis-inhibitory_8 | Moraxellaceae | *Acinetobacter* | unknown | 0.977 | 3.418 | 1.211 | 11.786 | 0.0006 | 0.008 |
| Smiliscasordida-inhibitory_4 | Oxalobacteraceae | unknown | unknown | 14.528 | 1.923 | 0.677 | 8.685 | 0.003 | 0.028 |
| Hemidactylumscutatum-inhibitory_18 | Oxalobacteraceae | *Janthinobacterium* | unknown | 2.329 | -2.308 | 0.760 | 8.925 | 0.003 | 0.027 |
| Ranamuscosa-inhibitory_37 | Comamonadaceae | unknown | unknown | 2.973 | 3.991 | 0.672 | 36.694 | 1.38E-09 | 1.22E-07 |
| Litorianannotis-inhibitory_54 | Enterobacteriaceae | unknown | unknown | 7.742 | 2.089 | 0.628 | 12.094 | 0.0005 | 0.007 |
| Hemidactylumscutatum-inhibitory_15 | Micrococcaceae | *Arthrobacter* | unknown | 4.068 | -3.337 | 0.754 | 16.423 | 5.07E-05 | 0.001 |

**Supplementary Table 13:** Differentially abundant antifungal microbes between animals based on *Bd* status and ANCOM analyses. Higher LogFoldChanges represent microbial taxa with greater abundance in *Bd+* individuals, and lower LogFoldChanges represent taxa with greater abundance in *Bd*– individuals**.**

| **Sample_ID** | **log_2_ Fold Change** | **Standard Error** | ***p*-value** | **Adjusted *p*-value** | **Test Statistic** |
| --- | --- | --- | --- | --- | --- |
| Craugastorcrassidigitus-inhibitory_91 | -0.4832 | 0.1346 | 0.0003 | 0.0243 | -3.5888 |
| Hemidactylumscutatum-inhibitory_15 | -0.5604 | 0.1612 | 0.0005 | 0.0366 | -3.47602 |
| Litoriaserrata-inhibitory_20 | -0.564 | 0.1465 | 0.0001 | 0.0087 | -3.85 |
| Ranamuscosa-inhibitory_48 | -0.6978 | 0.2058 | 0.0007 | 0.0496 | -3.39012 |
| Smiliscasordida-inhibitory_4 | 0.952 | 0.2369 | 0.0001 | 0.0044 | 4.01934 |
| Strabomantisbufoniformis-inhibitory_17 | 1.0069 | 0.2227 | 0.00E+00 | 0.0005 | 4.52055 |

**Supplementary Table 14.** Differentially abundant microbes without antifungal properties related to *Bd-*status based upon DESeq2 analyses. Microbes with a negative log_2_ fold change are more abundant in animals without *Bd* present, and ones with a positive log_2_ fold change are more abundant in animals with *Bd* found in the sample.

| **Family** | **Genus** | **Species** | **Base Mean** | **log_2_ FoldChange** | **Standard Error** | **Test Statistic** | ***p*-value** | **Adjusted *p*-value** |
| --- | --- | --- | --- | --- | --- | --- | --- | --- |
| Neisseriales | *Vogesella* | *fluminis* | 7.736 | 4.566 | 0.953 | 19.419 | 1.05E-05 | 0.016393 |
| Comamonadaceae | *Ideonella* | *paludis* | 2.086 | 4.277 | 1.034 | 22.504 | 2.10E-06 | 0.003931 |
| Comamonadaceae | *Rhizobacter* | unknown | 67.862 | 3.745 | 0.696 | 28.732 | 8.31E-08 | 0.000195 |
| Comamonadaceae | *Rhizobacter* | unknown | 317.617 | 6.075 | 0.635 | 78.146 | 9.57E-19 | 8.97E-15 |
| Comamonadaceae | *Rhizobacter* | unknown | 3.617 | 4.090 | 0.774 | 29.246 | 6.37E-08 | 0.000195 |
| Peptostreptococcaceae | *Romboutsia* | *sedimentorum* | 18.197 | 4.387 | 0.768 | 32.632 | 1.11E-08 | 5.22E-05 |

**Supplementary Table 15:** Differentially abundant microbes without antifungal properties related to *Bd-*status based upon ANCOM-BC analyses. Higher LogFoldChanges represent microbial taxa with greater abundance in *Bd+* individuals**.**

| **Sample ID** | **log_2_ Fold Change** | **Standard Error** | ***p*-value** | **Adjusted *p*-value** | **Test Statistic** |
| --- | --- | --- | --- | --- | --- |
| Acidovorax.defluvii | 0.903 | 0.193 | 0.000003 | 0.0006 | 4.670 |
| Aquitalea.denitrificans | 0.910 | 0.197 | 0.000004 | 0.0007 | 4.626 |
| Comamonas.jiangduensis | 0.574 | 0.147 | 0.000100 | 0.0188 | 3.891 |
| Comamonas.thiooxydans | 0.625 | 0.165 | 0.000156 | 0.0288 | 3.782 |
| Curvibacter.gracilis | 0.861 | 0.191 | 0.000007 | 0.0013 | 4.507 |
| DQ520187_s | 0.674 | 0.160 | 0.000025 | 0.0049 | 4.211 |
| EU089965_s | 1.426 | 0.244 | 5.05E-09 | 1.02E-06 | 5.845 |
| GQ850557_s | 0.638 | 0.155 | 0.000038 | 0.0073 | 4.118 |
| Hafnia.alvei | 0.830 | 0.222 | 0.000184 | 0.0337 | 3.740 |
| HQ111154_s | 1.090 | 0.247 | 0.000010 | 0.0019 | 4.423 |
| Ideonella.paludis | 1.143 | 0.189 | 1.36E-09 | 2.77E-07 | 6.060 |
| JZUE_s | 0.628 | 0.156 | 0.000054 | 0.0103 | 4.036 |
| Leptothrix.discophora | 1.404 | 0.227 | 6.27E-10 | 1.28E-07 | 6.184 |
| Polynucleobacter.sphagniphilus | 0.988 | 0.200 | 0.000001 | 0.0002 | 4.928 |
| Romboutsia.sedimentorum | 1.654 | 0.290 | 1.11E-08 | 2.23E-06 | 5.713 |
| Shigella.flexneri | 0.616 | 0.162 | 0.000139 | 0.0260 | 3.809 |
| Sphaerotilus.hippei | 0.749 | 0.190 | 0.000083 | 0.0156 | 3.936 |
| unk_S.18 | -0.875 | 0.230 | 0.000139 | 0.0260 | -3.810 |
| unk_S.25 | -0.581 | 0.155 | 0.000182 | 0.0334 | -3.743 |
| unk_S.30 | 0.717 | 0.177 | 0.000051 | 0.0098 | 4.050 |
| unk_S.9 | 3.372 | 0.380 | 7.07E-19 | 1.45E-16 | 8.874 |
| Vogesella.fluminis | 1.207 | 0.225 | 8.13E-08 | 1.63E-05 | 5.364 |
| Vogesella.mureinivorans | 1.153 | 0.247 | 0.000003 | 0.0006 | 4.663 |
